# Hybridization enables the fixation of selfish queen genotypes in eusocial colonies

**DOI:** 10.1002/evl3.253

**Published:** 2021-09-16

**Authors:** Arthur Weyna, Jonathan Romiguier, Charles Mullon

**Affiliations:** ^1^ Institut des Sciences de l'Evolution (UMR 5554) University of Montpellier, CNRS Montpellier 34000 France; ^2^ Department of Ecology and Evolution University of Lausanne Lausanne 1015 Switzerland

**Keywords:** Ant, caste determination, eusociality, genetic conflicts, hybridization, Hymenoptera, parasitism, reproductive system, social hybridogenesis

## Abstract

A eusocial colony typically consists of two main castes: queens that reproduce and sterile workers that help them. This division of labor, however, is vulnerable to genetic elements that favor the development of their carriers into queens. Several factors, such as intracolonial relatedness, can modulate the spread of such caste‐biasing genotypes. Here we investigate the effects of a notable yet understudied ecological setting: where larvae produced by hybridization develop into sterile workers. Using mathematical modeling, we show that the coevolution of hybridization with caste determination readily triggers an evolutionary arms race between nonhybrid larvae that increasingly develop into queens, and queens that increasingly hybridize to produce workers. Even where hybridization reduces worker function and colony fitness, this race can lead to the loss of developmental plasticity and to genetically hard‐wired caste determination. Overall, our results may help understand the repeated evolution toward remarkable reproductive systems (e.g., social hybridogenesis) observed in several ant species.

Eusociality is characterized by a striking division of reproductive labor between two castes: queens and workers (Crespi and Yanega 1995). Queens monopolize reproduction, while typically sterile workers specialize on other colony tasks such as foraging and tending to the brood. The sterility of workers initially seemed so inconsistent with natural selection that Darwin referred to eusociality as his “one special difficulty” (Darwin 1859, ch. 7). This apparent paradox was resolved in the 1960s with Hamilton's theory of kin selection (Hamilton 1964). Hamilton demonstrated that natural selection can favor eusociality when workers preferentially help relatives (who can transmit the same genetic material). In addition to laying the theoretical basis for the evolution of eusociality, Hamilton's work led to the insight that caste determination should be plastic to allow identical gene copies to be in workers and in the queen they help (Seger 1981). In line with this notion, the developmental fate of female larvae in many eusocial insects depends on environmental factors (Trible and Kronauer 2017), such as food quantity and quality (Brian 1956, 1973), temperature and seasonality (Brian 1974; Schwander et al. 2008) or signals emitted by adults of the colony (Penick and Liebig 2012; Libbrecht et al. 2013). Probably the most iconic example of such plasticity is found in honeybees where queens arise only from larvae reared in royal cells and fed with royal jelly. For long, this and many other empirical findings strengthened the idea that caste determination is under strict environmental control and largely free from genetic effects.

More recently, however, substantial genetic variation for caste determination has been described across a number of eusocial species (Winter and Buschinger 1986; Moritz et al. 2005; Hartfelder et al. 2006; Linksvayer 2006; Schwander and Keller 2008; Smith et al. 2008; Frohschammer and Heinze 2009; Schwander et al. 2010). This variation is thought to derive from caste‐biasing genotypes that bias the development of their carrier toward a particular caste (Moritz et al. 2005; Hughes and Boomsma 2008). Those genotypes that favor larval development toward the reproductive caste have sometimes been referred to as “royal cheats” as they cause the individuals that carry them to increase their own direct reproduction at the expense of other colony members (e.g., Anderson et al. 2008; Hughes and Boomsma 2008). The segregation of such royal cheats should depend on a balance between: (1) direct benefits from increased representation in the reproductive caste; and (2) indirect costs due to reduced worker production and colony productivity (Hamilton 1964). As highlighted by abundant theory, several factors can influence these benefits and costs and thus tip the balance for or against the evolution of royal cheats. For instance, low relatedness between larvae due to polyandry (when queens mate with multiple males) or polygyny (when colonies have multiple queens) increases competition between genetic lineages within colonies and thereby favors royal cheating (e.g., Reuter and Keller 2001). Conversely, selection against cheats is bolstered by low dispersal abilities and high within‐group relatedness (e.g., Hamilton 1964; Lehmann et al. 2008; Boomsma 2009), bivoltinism and asymetrical sex‐ratio (e.g., Trivers and Hare 1976; Seger 1983; Alpedrinha et al. 2014; González‐Forero 2015; Quiñones and Pen 2017), coercion (i.e., policing; Wenseleers et al. 2004; Dobata 2012), queen longevity and competition between queens (e.g., Queller 1994; Bourke and Chan 1999; Avila and Fromhage 2015), or where workers reproduce following queen death (Field and Toyoizumi 2020).

One intriguing factor that has been proposed to influence the cost of royal cheating is sperm parasitism, a behavior consisting in queens using the sperm of another species or lineage to produce hybrid workers (Linksvayer 2006; Anderson et al. 2008). Both morphological and genetic data suggest that this behavior is common in many ant species (e.g., in multiple *Temnothorax* populations, the majority of queens were found to produce some hybrid workers; Douwes and Stille 1991; Umphrey 2006 and Feldhaar et al. 2008 for reviews). In these species, sperm parasitism results in hybrid larvae that rarely, if ever, develop as fertile queens and rather become sterile workers (presumably due to genetic incompatibilities between parental lineages; Feldhaar et al. 2008; Trible and Kronauer 2017). Such hybrids should therefore be impervious to genetic caste‐biasing effects and thus provide a reliable source of workers. In principle, this alternative supply of workers may reduce the indirect cost of royal cheats and hence favor their evolution (Anderson et al. 2008). But beyond these broad‐brush predictions, the effect of sperm parasitism on the segregation of royal cheats remains poorly understood.

Here, we develop a mathematical model to explore the evolution of genetic caste determination via royal cheats when queens can hybridize to produce workers. In particular, we assess the effects of key factors on the evolutionary dynamics of caste determination, such as polyandry and queen parthenogenesis (when queens have the ability to produce daughters asexually), as well as their interactions with potential costs and benefits of hybridization, for instance, owing to hybrid incompatibilities or hybrid vigor.

## The Model

**Figure 1:**
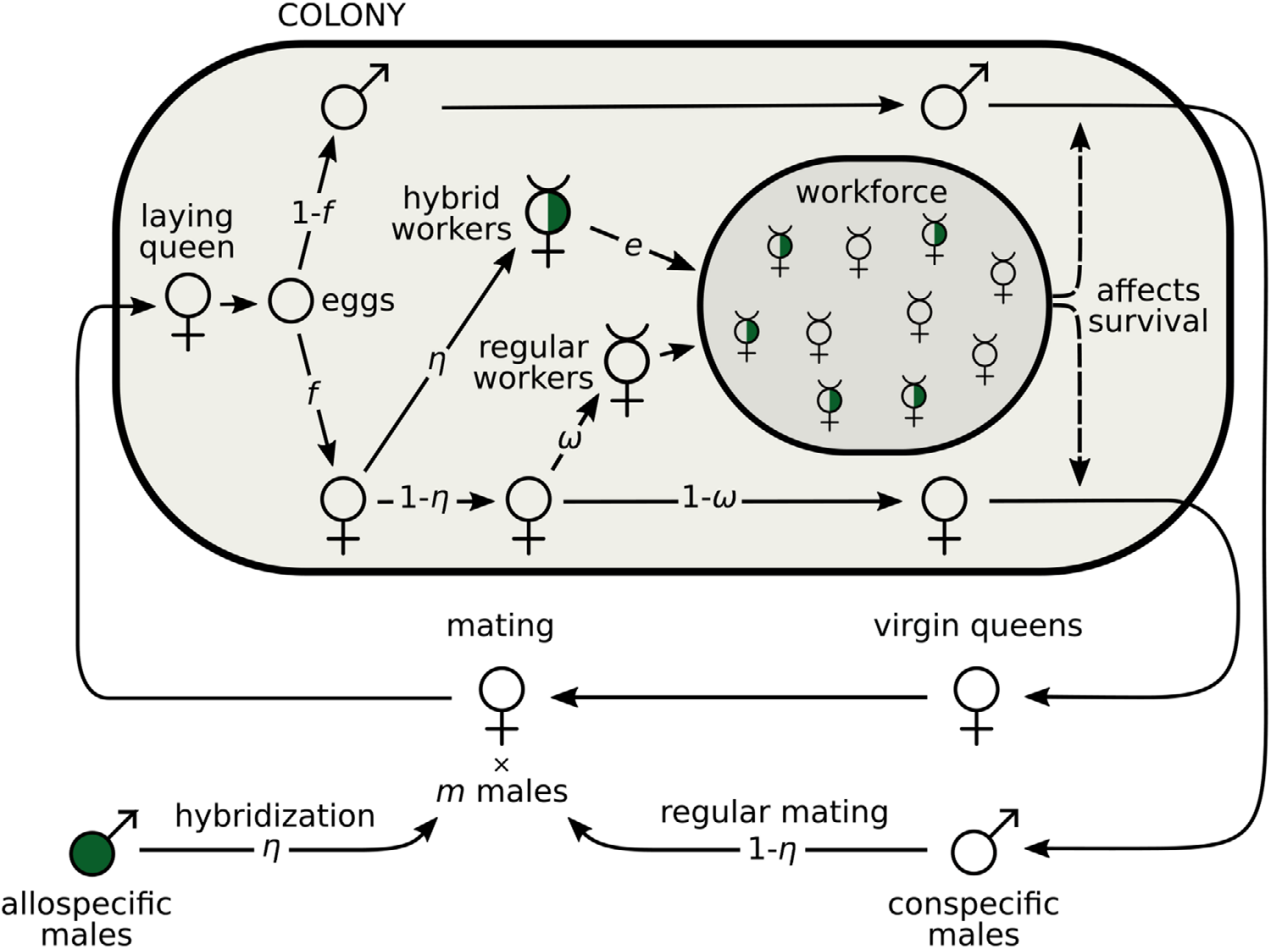
The life cycle of an annual eusocial with hybridization and sperm parasitism. At each generation, the life‐cycle begins with virgin queens mating with m males, each of which has a probability η to be allospecific and 1−η to be conspecific. After mating, a queen founds a colony and starts producing eggs. Hybrid female eggs (with allospecific paternal origin) all develop into workers. Regular female eggs (with conspecific paternal origin) develop into workers with probability ω and into queens otherwise. The variable η thus captures the tendency of queens to hybridize and parasitize sperm, while ω controls caste determination.

We consider a large population of annual eusocial haplodiploids with the following life‐cycle (Fig. 1). First, virgin queens mate with a fixed number m∈{1,2,…} of males. Each of these mates can either be an allo‐ (with probability η) or a con‐specific male (with complementary probability 1−η). Once mated, queens found monogynous colonies (i.e., one queen per colony) and lay a large number of eggs. A proportion f of these eggs are diploid (and develop into females) and (1−f) are haploid (and develop into males). Assuming random egg fertilization, a queen therefore produces on average fη hybrid and f(1−η) nonhybrid females. We assume that a hybrid female can only develop as a worker, while a nonhybrid female can either develop as a worker (with probability ω) or as a queen (with complementary probability 1−ω). Overall, a colony thus consists of fη hybrid and f(1−η)ω nonhybrid sterile workers, as well as f(1−η)(1−ω) virgin queens and (1−f) males that are available for reproduction at the next generation.

If only virgin queens and males can reproduce, their reproductive success depends on the workforce of their colony of origin. Specifically, we assume that the probability that a sexual reaches the mating pool increases linearly with the total number of workers in the colony, combining hybrid and nonhybrid workers (we show later that our results do not change qualitatively when the increase is nonlinear). We nonetheless allow for differential contribution to the workload between hybrid and nonhybrid workers, with the contribution of hybrid workers weighted by a parameter e≥0 (so that the effective workforce of a colony is efη+f(1−η)ω). When e=1, hybrid workers have the same working efficiency as nonhybrid workers. By contrast, when e<1, hybrid workers are less efficient, for instance, due to outbreeding depression. This can also reflect other potential costs associated with hybridization, such as the production of sterile or nonviable hybrid queens (Feldhaar et al. 2008). Conversely, when e>1, hybrid workers outperform regular workers, due, for example, to hybrid vigor (Umphrey 2006).

## Results

### HYBRIDIZATION AND SPERM PARASITISM, EVEN COSTLY, CAN LEAD TO THE FIXATION OF ROYAL CHEATS AND THE COMPLETE LOSS OF INTRASPECIFIC WORKERS

We first investigate the evolution of caste determination by allowing the probability ω that a larva develops as a worker to vary. We assume that this probability is under individual genetic control (i.e., the future caste of a female larva depends only on its own genotype) and that it evolves via random mutations with weak additive phenotypic effects (Appendix A for details on our methods). Mutational effects are unbiased so a new mutation is equally likely to increase or decrease the tendency ω of becoming a worker. Those mutations that decrease ω can be considered as more selfish as they increase the likelihood that their carriers develop into queens at the expense of other individuals of the same colony. Following the terminology of Hughes and Boomsma (2008), we thus refer to mutations decreasing ω as royal cheats. As a baseline, we consider the case where queens mate with a large number of males (i.e., m→∞) and where hybridization is fixed at a given level (e.g., η is the proportion of allo‐specific males in the pool of mates from which females choose randomly).

Our analyses (Appendix B.1.1) reveal that the probability for a larva to develop as a worker evolves toward a unique and stable equilibrium,

(1) $$\omega^{*}=\frac{1}{3}-e\frac{2\eta }{3(1-\eta )}.$$

**Figure 2:**
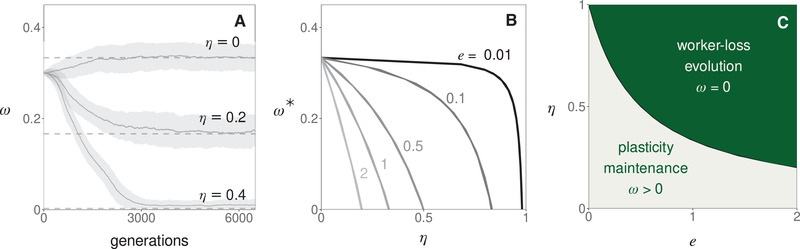
The fixation of royal cheats and evolution of intraspecific worker‐loss. (A) Evolution of the probability ω that a female larva develops into a worker in a simulated population when queens mate with a large number of males (polyandry, m→∞) and the proportion of allospecific males η is fixed (top η=0; middle η=0.2, bottom η=0.4; other parameters: e=1, Appendix A.3 for details on simulations). Plain lines (and surrounding gray areas) show the population average ω (and its standard deviation). Dashed lines show the predicted equilibrium (from eq. 1). (B) Equilibrium of ω as a function of hybridization η and the efficiency of hybrid workers e (from eq. 1). (C) Parameter combinations leading to the evolution of complete worker‐loss (i.e., ω→0, in green, corresponding to η≥1/(1+2e), which is found by substituting eq. 1 into $\omega^{*}\leq 0$).

To interpret this equation (1), consider first the case where hybridization is costless (e=1). Equation (1) then tells that in the absence of hybridization (η=0), a larva will develop into a worker with a probability of 1/3 at equilibrium (in line with previous models that ignore hybridization, e.g., Reuter and Keller 2001, Appendix B.1.4 for connection). But as hybridization increases (η>0), royal cheating is increasingly favored and larvae become increasingly likely to develop as queens rather than workers (i.e., $\omega^{*}<1/3$, Fig. 2A). In fact past a threshold of hybridization (η≥1/3), the population evolves toward a complete loss of nonhybrid workers via the fixation of increasingly caste‐biasing royal cheats alleles (ω→0). In this case, nonhybrid females eventually all develop into queens that rely on sperm parasitism to produce workers.

Equation (1) also shows that the performance of hybrid workers relative to nonhybrids, e, modulates the effect of hybridization on the evolution of caste determination (Fig. 2B). As a result, royal cheating and worker‐loss evolution are facilitated when hybrids outperform regular workers (e>1) but hindered otherwise (e<1). Nevertheless, even when hybridization is extremely costly (0<e≪1), complete worker‐loss can evolve (Fig. 2C).

### WORKER‐LOSS READILY EMERGES FROM THE COEVOLUTION OF GENETIC CASTE DETERMINATION AND SPERM PARASITISM, DRIVEN BY INTRACOLONIAL CONFLICT

The above analysis indicates that intraspecific worker‐loss can evolve when queens have a sufficiently high tendency to hybridize. This raises the question of whether such tendency is also subject to selection. To answer this question, we allow the probability η that a queen's mate is allospecific to coevolve with caste determination (ω). We assume that this probability η is under individual queen control (i.e., it depends only on a queen's genotype) and like caste determination, evolves via rare mutations with weak additive phenotypic effects (Appendix A for details).

**Figure 3:**
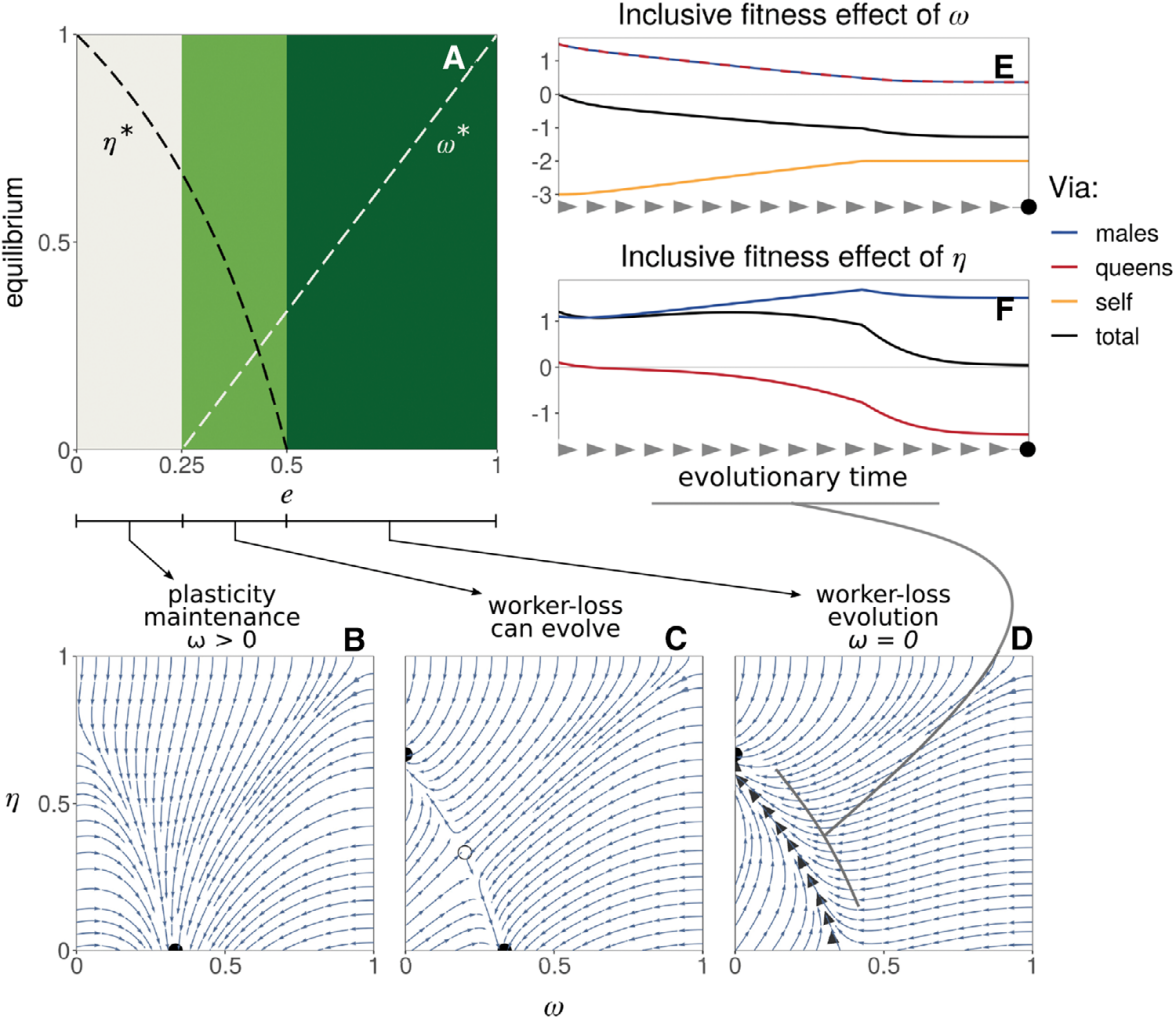
The coevolution of caste determination and sperm parasitism. (A) Evolutionary equilibria (for η in black and ω in white) as a function of hybrid worker efficiency e (eq. B6 in Appendix B.1.2 for details). These equilibria, however, are evolutionary repellors (eq. B7 in Appendix B.1.2). As a result, three types of coevolutionary dynamics are possible depending on e as illustrated in panels (B)–(D) (from eq. B5). These panels show examples of phenotypic trajectories when worker‐loss: Panel (B) never evolves (e=0.1); Panel (C) can evolve depending on initial conditions (e=0.4); Panel (D) always evolves (e=0.7). Black filled circles indicate the two evolutionary end‐points: hybridization avoidance with developmental plasticity (ω=1/3 and η=0 in B and C) or worker‐loss with hybridization (ω=0 and η=2/3 in C and D). Empty circle in (C) shows the internal unstable equilibrium (eq. B6). Thick grey arrow heads in (D) represent the trajectory of a population starting from ω=1/3 and η=0 and evolving to worker‐loss. (E) Fitness effects of caste determination ω in a mutant larva via itself (in orange), related queens (red), and related males (blue) along the trajectory leading to worker‐loss shown in panel (D) (total selection in black, Appendix B.1.3 for derivation). We see that negative fitness effects via self (orange line) lead to a total selection effect that is negative (black line). This indicates that mutant larvae with increasingly small values of ω are selected because these values increase larvae's direct fitness (by increasing the probability that they develop into queens). (F) Fitness effects of hybridization η in a mutant queen, via its sons (blue) and daughter queens (red) along the trajectory leading to worker‐loss shown in panel (D) (total selection in black). Positive total selection (in black) is mostly due to an increase of fitness via males (in blue). This says that mutant queens with increasingly large values of η are selected because this increases their reproduction, especially via males.

We find that depending on the efficiency e of hybrid workers, the coupled evolutionary dynamics of hybridization η and caste determination ω lead to an evolutionary arms race with one of two contrasted outcomes (Appendix B.1.2 for analysis). When e is small (e≤1/4, Fig. 3A gray region), the population evolves hybridization avoidance (η→0) while the probability ω to develop as a worker stabilizes for its baseline equilibrium ($\omega^{*}=1/3$, Fig. 3B). By contrast, when hybrid workers are at least half as efficient as regular workers (e≥1/2, Fig. 3A, dark green region), intraspecific worker‐loss evolves (ω→0) and hybridization stabilizes at an intermediate equilibrium ($\eta^{*}=2/3$, Fig. 3D). When hybrid worker efficiency is intermediate (1/4<e<1/2, Fig. 3A, light green region), the population evolves either hybridization avoidance or intraspecific worker‐loss depending on initial conditions (Fig. 3C), with worker‐loss favored by high initial tendency η of queens to hybridize. In sum, provided four hybrid workers are at least as good as one regular worker (e>1/4), the coevolution of genetic caste determination and hybridization can lead to worker‐loss in our model.

To better understand the forces at play in the emergence of worker‐loss, we further used a kin‐selection approach to decompose the invasion fitness of mutant alleles into the sum of: (1) their direct fitness effects on the reproductive success of the individuals that express them; and (2) of their indirect fitness effects on other related individuals that can also transmit them (Taylor and Frank 1996, Appendix B.1.3 for details). Starting with a population at the baseline equilibrium in absence of hybridization (ω=1/3, η=0), we tracked these different fitness effects along a typical evolutionary trajectory that leads to worker‐loss (black arrow heads, Fig. 3D) for alleles that influence the tendency of a larva to develop as a worker (Fig. 3E) and of a queen to hybridize (Fig. 3F).

Our kin selection analysis reveals that alleles that increase hybridization in queens are selected because they allow queens to increase the number of sexuals produced by their colony (especially via males, blue curve, Fig. 3F). This is because the baseline tendency ω to develop as a worker that evolves is optimal from the point of view of a gene in a larvae, but sub‐optimal from the point of view of a gene in a queen who would benefit from a larger workforce. Hybridization by queens evolves to rectify this and align colony composition with the interests of the queen. Simultaneously, as queens evolve greater hybridization and augment their workforce with hybrids, genes in nonhybrid larva have an increasing interest for their carriers to develop as queens rather than workers (Fig. 3E). These two selective processes via queens and larvae fuel one another in an evolutionary arms race whose endpoint is complete intraspecific worker‐loss. Our decomposition of fitness effects thus shows that the loss of nonhybrid workers evolves in our model due to within‐colony conflicts over colony composition. In fact, our results suggests that worker‐loss emerges because hybridization allows queens to control the production of workers in their colony, while nonhybrid larvae lose their tendency to develop as workers to promote their own reproduction via the fixation of royal cheats.

### WORKER‐LOSS IS IMPAIRED BY LOW POLYANDRY BUT FACILITATED BY ASEXUAL REPRODUCTION

So far, we have assumed that queens mate with a large, effectively infinite, number of males. By increasing relatedness within the brood, low polyandry (2≤m≪∞), and monandry (m=1) mediate within‐colony conflicts and therefore should be relevant to the evolutionary arms race leading to worker‐loss (Anderson et al. 2008; Schwander et al. 2010). To test this, we investigated the effect of mate number m on the coevolution of ω and η (Appendix B.2.1 for details).

**Figure 4:**
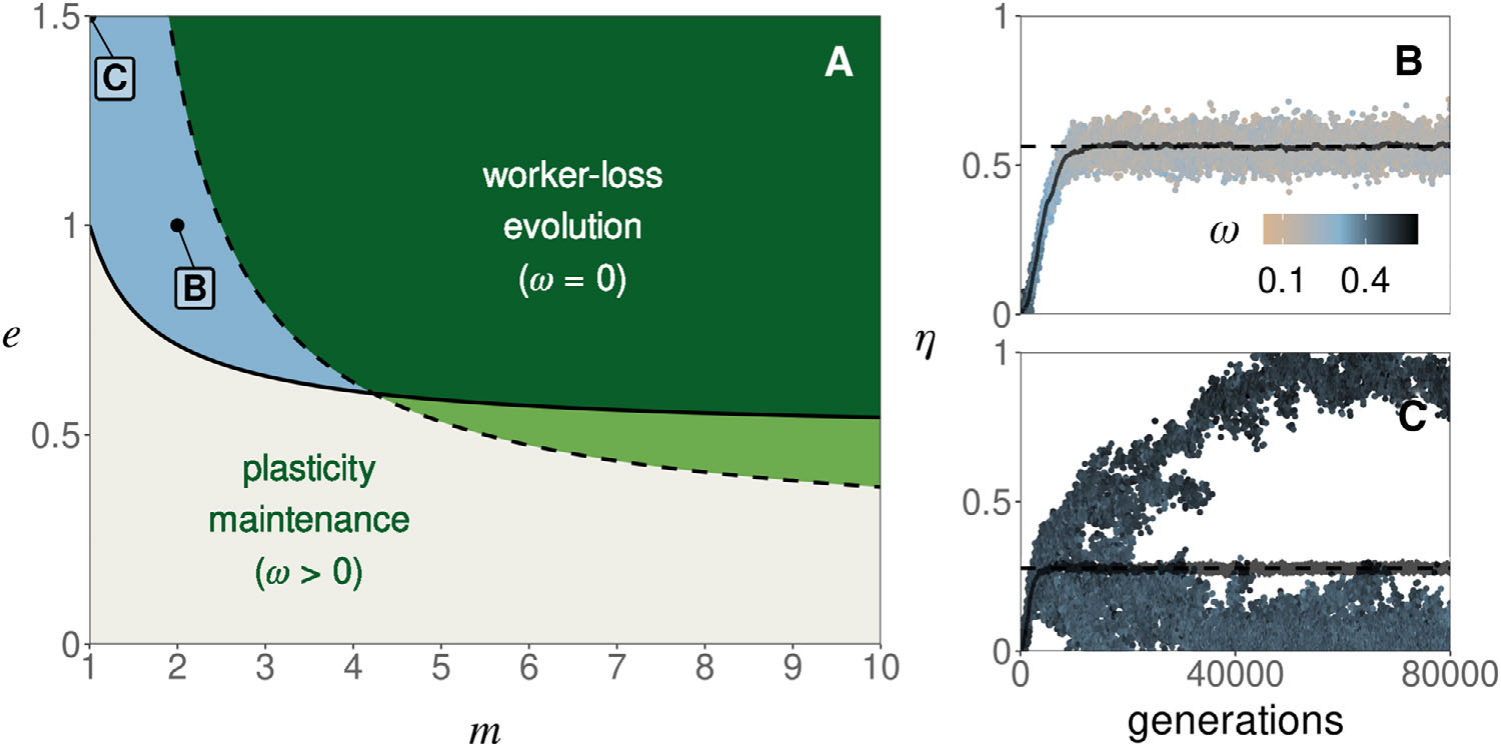
The effects of monandry and low polyandry. (A) Outcome of selection as a function of mate number m and hybrid worker efficiency e. Over the dashed line, worker‐loss is a stable equilibrium (i.e., a population with traits ω=0 and η=2/3 cannot be invaded, eq. B16 in Appendix B.2.1). Over the plain line, hybridization can invade when rare (i.e., η=0 is unstable, eq. B18 in Appendix B.2.1). Below both lines (gray region), plasticity in caste determination is maintained (as in Fig. 3B). Over both lines (dark green region), hybridization and worker‐loss evolve (as in Fig. 3D). In the light green region, worker‐loss evolve for some initial conditions (as in Fig. 3C). In the blue region, there exists an internal attractor equilibrium (i.e., the population converges toward a phenotype $0<\eta^{*}<1$ and $0<\omega^{*}<1$) that is either uninvadable (for 2≤m≤4, see, e.g., panel B) or invadable leading to polymorphism (for m=1, see, e.g., panel C). (B) Evolution toward an uninvadable phenotype in a simulated population (when e=1 and m=2). Each dot represents the value of η of one of 20 haplotypes randomly sampled every 100 generation in a simulated population of 10,000 queens (Appendix A.3 for details on simulations). The color of each dot gives the value of ω of the associated haplotype (legend). The horizontal dashed line represents the predicted equilibrium (from Fig. S1). The gray line represents the mean value of η across the simulation. (C) Evolution toward an invadable phenotype and the emergence of polymorphism in a simulated population (when e=1.5 and m=1, other parameters and figure legend: same as B).

We find that as the number m of mates decreases, the conditions for intraspecific worker‐loss emergence become more restrictive. Specifically, the threshold of hybrid worker efficiency e above which worker‐loss always evolves increases as polyandry decreases (as m→1, Fig. 4A, dark green region). In addition, when the number of mates is low (m≤4), evolutionary dynamics do not necessarily lead to either complete worker‐loss or hybridization avoidance. For intermediate values of e (Fig. 4A, blue region) the population actually converges to an intermediate state where queens partially hybridize ($0<\eta^{*}<1$) and larvae retain developmental plasticity ($0<\omega^{*}<1$, Fig. 4B, Appendix B.2.1 and Fig. S1 for analysis). Under monandry (m=1) the evolution toward such intermediate state always happens when hybrid workers outperform regular workers (e>1, Fig. 4A, blue region).

In the special case of monandry and overperforming hybrid workers (m=1 and e>1), our mathematical analysis further shows that partial hybridization and larval plasticity is not evolutionary stable (Appendix B.2.1, Figs. S1 and S2). Rather, the population experiences disruptive selection that should favor the emergence of polymorphism. To test this, we performed individual‐based simulations under conditions predicted to lead to polymorphism (Fig. 4C). These show the emergence and long‐term coexistence of two types of queens: one that hybridizes with low probability (and reproduces via both males and queens); and another that mates almost exclusively with allospecific males and thus reproduces mostly via males (because m=1, these queens only produce hybrid workers and males). Beyond this special case, the evolution of worker‐loss is impeded by low polyandry and impossible under monandry in our model. This is because with a low number of mates, a queen runs the risk of being fertilized by only one type of male. Under complete worker‐loss (when the population is fixed for ω=0), a queen mated to only conspecific males produces only larvae destined to be queens but no workers to ensure their survival and thus has zero fitness.

Our finding that monandry inhibits the emergence of worker‐loss contrasts with the observation that several ant species, notably of the genus *Cataglyphis*, lack nonhybrid workers and rely on sperm parasitism for workers in spite of being mostly monandrous (Kuhn et al. 2020). One potential mechanism that could have allowed such evolution is thelytokous parthenogenetic reproduction by queens, whereby queens can produce daughters clonally. This reproduction mode, which is common in eusocial Hymenoptera (Rabeling and Kronauer 2013) and in particular in *Cataglyphis* (Kuhn et al. 2020), could allow queens fertilized exclusively by allospecific males to nevertheless produce queens via parthenogenesis. To investigate how thelytokous parthenogenesis influences the evolution of caste determination, we extend our model so that a fraction c of the female progeny of queens is produced parthenogenetically (Appendix B.2.2 for details). We assume that larvae produced in such a way are equivalent to nonhybrid larvae: they develop into workers with a probability ω determined by their own genotype (which in this case is the same as their mother's genotype) and if they develop into workers, they have the same working efficiency as nonhybrid workers (i.e., there is no direct cost or benefit to parthenogenesis).

**Figure 5:**
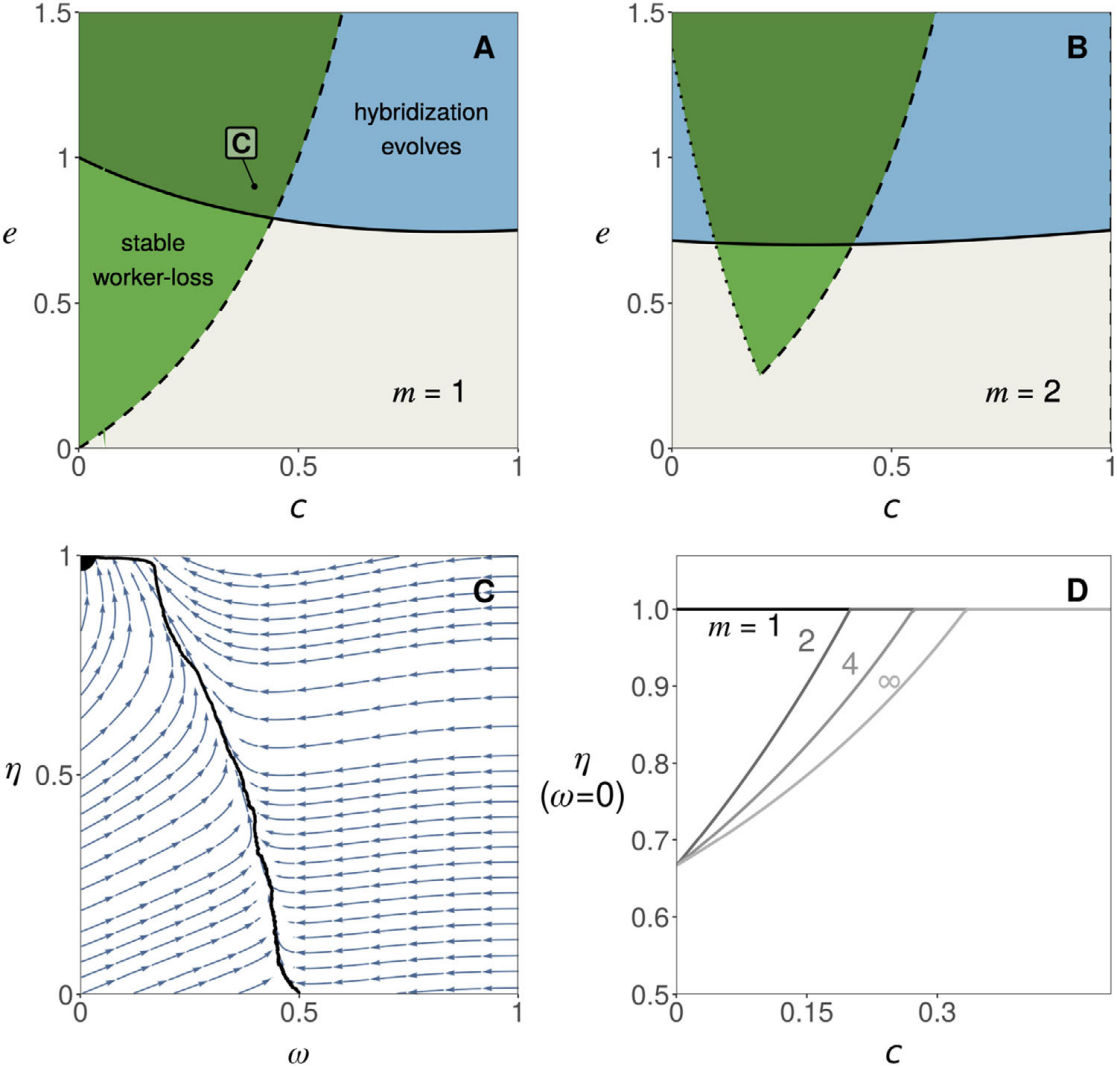
The influence of thelytokous parthenogenesis. (A) and (B) Invasion analysis as a function of parthenogenesis c and hybrid worker efficiency e (with m=1 in A and m=2 in B). In the region over the plain line, hybridization can invade when rare (i.e., η=0 is unstable, eq. B23). In the region over the dashed line (in A) or framed by the dotted and dashed lines (in B), worker‐loss is a stable equilibrium (i.e., a population at equilibrium for η and with ω=0 cannot be invaded, Appendix B.2.2, eqs. B25 and B26 for details). In the dark green region, selection thus favors both the evolution of hybridization and the maintenance of worker‐loss (e.g., panel C). In the light green region, worker‐loss can evolve only for some initial conditions (as in Fig. 3C). (C) Phenotypic trajectories leading to worker‐loss (when e=0.9, c=0.4, and m=1). Arrows show the direction of evolution favored by selection. Black filled circles indicate the evolutionary end‐point. The black line shows the average trait values of a simulated population starting at (ω=1/2,η=0). In this example, selection leads to a state where worker‐loss (ω=0) is coupled with complete hybridization (η=1). (D) Level of hybridization η favored by selection when worker‐loss has evolved (ω=0) as a function of parthenogenesis c. This shows that worker‐loss is always associated to complete hybridization (η=1) under monandry (m=1) and if c≥(m−1)/(3m−1) under polyandry (m>1) (Appendix B.2.2, eq. B24, for details).

The coevolutionary dynamics of caste determination and hybridization with parthenogenesis are in general too complicated to be tractable. We could nonetheless gain insights into worker‐loss evolution by performing an invasion analysis, asking (1) when is worker‐loss (ω=0) evolutionary stable (so that a population where intraspecific workers have been lost cannot be invaded by a genetic mutant with developmental plasticity)? And (2) when can hybridization evolve when absent in the population (i.e., when is η=0 evolutionary unstable)? When these two conditions are met, evolution will tend to favor the emergence and maintenance of worker‐loss (e.g., as in Fig. 3D). We thus studied when conditions (1) and (2) above are both true in terms of parthenogenesis c, as well as hybrid workers efficiency e and mate number m. This revealed that parthenogenesis has a nonmonotonic relationship with worker‐loss evolution (Fig. 5A and B). As parthenogenesis increases from zero, worker‐loss evolution is initially favored, especially under monandry (as expected; e.g., Fig. 5C; see eq. B26 in the Appendix for details). But past a threshold of parthenogenesis, the conditions leading to worker‐loss become increasingly stringent until such evolution becomes impossible (see eq. B25 in the Appendix for details). This is because as parthenogenesis increases, the relatedness among a queen and larvae of the same colony also increases. The conflict between them, which fuels the evolution of worker‐loss, therefore abates until it is no longer advantageous for a larva to preferentially develop as a queen.

We additionally computed the level of hybridization favored by selection when the population has evolved worker‐loss (and this is an evolutionarily stable state). We find that hybridization increases as queens mate with fewer males and as parthenogenesis increases (Fig. 5D), so much so that selection can lead to complete hybridization (η=1, e.g., Fig. 5C). As a result, there exists a range of intermediate values of parthenogenesis for which worker‐loss evolves in association with a complete loss of intraspecific matings, that is, queens never mate with males of their own species or lineage. These males are nevertheless still being produced in our model (as the primary sex ratio is such that f<1).

## Discussion

In sum, our analyses indicate that worker‐loss readily evolves when queens can hybridize with a lineage of males by whom fertilization leads to the production of workers. This evolution in our model occurs through a sequence of substitutions of alleles that increasingly bias the development of their carrier toward the queen caste, that is, “royal cheats”. Hybridization, or sperm parasitism, allows royal cheats to fix in the population by providing a way for colonies to compensate for the reduced workforce. In fact, when queens are capable of recognizing genetic differences among males and when royal cheats are present in the population, selection favors hybridization by queens to regain control over caste allocation in their colony. This in turn promotes greater cheating by larvae, which favors greater hybridization by queens and so on. This evolutionary arms race, fueled by intracolonial conflicts, eventually leads to complete intraspecific worker‐loss: a state where larvae have lost their developmental plasticity and develop as workers or queens depending only on whether they are the product of hybridization or not, respectively.

### MODEL LIMITATIONS

Of course, our analyses are based on several idealized assumptions. In particular, we assumed that the probability for larvae to develop as workers is under complete larval genetic control. Typically the developmental fate of female larvae also depends on various environmental factors created by adult colony members, such as food quality and quantity (Brian 1956; Trible and Kronauer 2017), or mechanical (Penick and Liebig 2012) and chemical (Schwander et al. 2008; Penick et al. 2012) stimuli. The conclusions of our study apply as long as these environmental effects are held constant (or evolve more slowly than genetic caste determination). In this case, worker‐loss would emerge via royal cheats that modify larval developmental reaction norm to environmental effects in such a way that their carriers are more likely to develop as queens (Hughes and Boomsma 2008; Wolf et al. 2018). We also assumed that caste determination and hybridization evolve via rare mutations with weak additive effects at a single locus. These assumptions, which are typical to adaptive dynamics and kin selection approaches, have been extensively discussed elsewhere in a general context (Frank 1998; Rousset 2004; Geritz and Gyllenberg 2005; Dercole and Rinaldi 2008). In particular, all our results extend to the case where traits are determined by many genes and/or many co‐segregating alleles, provided genetic variance in the population remains small (e.g., Charlesworth 1990; Iwasa et al. 1991; Abrams et al. 1993; Mullon and Lehmann 2019). In cases where mutations have large additive or dominance effects, we expect more complex evolutionary dynamics, such as genetic polymorphism. These dynamics can nonetheless be straightforwardly investigated with the recurrence equations we derived (eq. A4 in Appendix). However, our model cannot accommodate potential interaction effects among loci (i.e., epistasis). If a quantitative genetics analysis in *Temnothorax curvispinosus* supports that caste determination is influenced by additive effects in this species (Linksvayer 2006), only epistatic effects were found in *Pogonomyrmex rugosus* (Schwander and Keller 2008). It would therefore be relevant in the future to allow for a more complex genetic basis of caste determination, including epistasis (in particular, in the context of the evolution of unorthodox reproductive systems, see next section). Another important assumption we made is that hybrid larvae do not develop into fertile queens, for instance owing to hybrid incompatibilities (Trible and Kronauer 2017). If fertile hybrid queens are produced regularly, evolution toward worker‐loss like in our model is less likely to happen as hybrids no longer make a reliable source of workers. In ants at least, the idea that hybrid queens are rarely fertile is supported by the contrast between high frequency of interspecific mating on one hand, and weak genetic signals of interspecific gene flow on the other (Umphrey 2006; Feldhaar et al. 2008). Finally, we focused in the main text on the case where colony productivity increases linearly with workers (i.e., the probability that a sexual survives until reproduction increases linearly with the number of workers). More realistically, the gain in productivity brought by one additional worker is likely to decrease with increasing workforce (Nonacs and Tobin 1992; Reuter and Keller 2001). Such diminishing returns tend to favor cheating because the indirect benefit of developing into a worker gets smaller as colony size increases (e.g., Reuter and Keller 2001; Field and Toyoizumi 2020). In line with this, we find that worker‐loss evolves even more easily under diminishing compared to linear returns (Appendix B.2.3 and fig. S3).

### AN ADAPTIVE PATH TO UNORTHODOX REPRODUCTIVE SYSTEMS?

Our result that sperm parasitism favors the emergence of worker‐loss via the fixation of royal cheats may be relevant to unorthodox reproductive systems found in ants. Of particular interest is social hybridogenesis, whereby females produced through regular intralineage mating or thelytokous parthenogenesis develop into queens, while workers emerge from eggs fertilized by allospecific males (Helms Cahan et al. 2002; Helms Cahan and Vinson 2003; Anderson et al. 2006; Romiguier et al. 2017; Lacy et al. 2019; Kuhn et al. 2020). Such a striking system was first described just two decades ago in *Pogonomyrmex* harvester ants (Helms Cahan et al. 2002), and has since been found in several species spread across four genera (Helms Cahan et al. 2002; Helms Cahan and Vinson 2003; Romiguier et al. 2017; Lacy et al. 2019; Kuhn et al. 2020). If these observations suggest that social hybridogenesis has evolved independently multiple times, the evolutionary origins of this complex system remain poorly understood (Anderson et al. 2008; Schwander et al. 2010; Lavanchy and Schwander 2019). One early suggestion is based on the hypothesis that worker development requires the combination of co‐adapted alleles at key loci (i.e., requires epistatic interactions; Helms Cahan and Keller 2003). According to this theory, worker‐loss in hybridogenetic lineages would have originated in the random loss of such combinations during episodes of ancestral hybridization. Present hybridization would then have evolved to restore genetic combinations and epistatic interactions in F1‐hybrids allowing for worker development.

Here, we have shown mathematically that social hybridogenesis could also result from additive genetic effects on caste development and queen‐larvae conflicts within colonies. This theory, previously described verbally in Anderson et al. (2006, 2008), may help explain the multiple convergence toward social hybridogenesis because virtually every sexual eusocial species should experience queen‐larvae conflicts over caste investment. Furthermore, because this path to social hybridogenesis does not depend on changes in the sympatric species whose sperm is parasitized, our model is relevant to both cases of asymmetrical (where the sympatric species produces workers through regular sex, as, e.g., in *Solenopsis xyloni*; Helms Cahan and Vinson 2003) and symmetrical social hybridogenesis (where the sympatric species also produces workers via hybridization, as, e.g., in *Pogonomyrmex* harvester ants; Anderson et al. 2006).

Our model may also be relevant to other unorthodox systems of reproduction such as those found in populations of *Wasmannia auropunctata* (Fournier et al. 2005), *Vollenhovia emeyri* (Ohkawara et al. 2006), or *Paratrechina longicornis* (Pearcy et al. 2011). As with some forms of social hybridogenesis, queens of these systems produce their reproductive daughters via female parthenogenesis and their workers via sex with genetically distant males. In contrast to social hybridogenesis, however, these males belong to a divergent all‐male lineage maintained by male clonality. This is further accompanied with a complete absence of arrhenotokous males (i.e., queens never make hemiclonal haploid sons, as shown in *W. auropunctata*; Rey et al. 2013). When queens are able to produce daughters parthenogenetically in our model, evolution can lead to a state where worker‐loss is coupled with a complete absence of intralineage mating (i.e., η=1, Fig. 5C and D). In this state, arrhenotokous males represent a genetic dead‐end, laying the basis for their disappearance. To investigate these systems in more detail, it would be interesting to extend our model to consider the evolution of female parthenogenesis and male clonality.

Our formal approach is especially useful in a context where hybrid vigor in workers has been raised to explain the evolutionary origin of social hybridogenesis and other hybridization‐dependent systems (Julian and Cahan 2006; Umphrey 2006; Anderson et al. 2008; Feldhaar et al. 2008; Schwander et al. 2010). According to this argument, selection favored hybridization because hybrid workers are more efficient, more resilient, or better suited to exploit marginal habitats than regular workers. But in spite of much effort, empirical evidence supporting hybrid vigor in workers is still lacking (Ross and Robertson 1990; James et al. 2002; Julian and Cahan 2006; Feldhaar et al. 2008). Further challenging this view, we have shown here that hybrid vigor is not necessary to the evolution of hybridization‐dependent reproductive systems. In fact, our results demonstrate that these systems can easily evolve even when hybridization is costly due to pre‐ and postzygotic barriers (i.e., when e<1, e.g., because hybridization leads to an inefficient workforce due to hybrid incompatibilities in workers; or increased efforts in mate‐finding and mating, Maroja et al. 2014; or the production of nonviable or infertile hybrid queens, Umphrey 2006; Feldhaar et al. 2008). In contrast to previous suggestions (Anderson et al. 2008), our model thus indicates that hybridization‐dependent reproductive systems can emerge among species that have already substantially diverged, and can be maintained even with further accumulation of hybrid incompatibilities.

More generally, our results suggest that natural selection can lead to an association between hybridization and caste determination. To date, such associations have been reported in only 18 distinct ant species or populations (Helms Cahan et al. 2002; Helms Cahan and Vinson 2003; Fournier et al. 2005; Anderson et al. 2006; Ohkawara et al. 2006; Pearcy et al. 2011; Romiguier et al. 2017; Lacy et al. 2019; Kuhn et al. 2020). But this rarity may be due—at least partly—to the difficulty with describing these systems (which in particular requires sampling and genotyping both queens and workers of the same populations, Helms Cahan et al. 2002). For instance, studies specifically testing for social hybridogenesis discovered five new cases of this reproductive system in *Cataglyphis* (out of 11 species tested, Kuhn et al. 2020) and three in *Messor* (out of 9, Romiguier et al. 2017). These considerations, together with our results, support the notion that currently known cases likely represent only a small fraction of extant eusocial systems relying on hybridization (Helms Cahan et al. 2002; Lavanchy and Schwander 2019).

### FACTORS PROMOTING THE EVOLUTION OF INTRASPECIFIC WORKER‐LOSS

In addition to showing that hybrid vigor is not necessary for the emergence of intraspecific worker‐loss, our model highlights several factors that can facilitate such evolution. The first of these is polyandry, which favors sperm parasitism and worker‐loss by minimizing the risks associated with hybridization. Interestingly, even though polyandry is generally rare in social insects (Strassmann 2001; Hughes et al. 2008), meaningful exceptions are found in *Pogonomyrmex* (Rheindt et al. 2004) and *Messor* (Norman et al. 2016) harvester ants, two taxa where social hybridogenesis has evolved multiple times (Anderson et al. 2006; Romiguier et al. 2017). Although the number of males a queen mates with is fixed in our model, it is conceivable that this number also responds to hybridization, leading polyandry and hybridization to coevolve. Indeed as low levels of polyandry represent a risk for out‐breeding queens, we can expect selection to favor queen behaviors that increase their number of mates. This would in turn allow for greater levels of hybridization, which would increase selection on polyandry and so on. We therefore expect that the coevolution between polyandry, hybridization, and caste determination further promotes the emergence of worker loss. For species that are fixed for strict (or close to) monandry, our model shows that worker‐loss can evolve when queens have the ability to reproduce via thelytokous parthenogenesis as it allows interspecifically mated queens to nevertheless produce daughter queens. This supports the notion that thelytoky has been important for the convergent evolution of social hybridogenesis in the (mostly) monandrous *Cataglyphis* ants (Kuhn et al. 2020).

Although not considered in our study for simplicity, another factor that can minimize the risks associated with hybridization in monandrous species is polygyny, whereby related queens form multi‐queen nests. Such social organization allows both intra‐ and interspecifically mated queens to be part of the same colony, which can then produce both queens and workers. Polygyny should therefore further facilitate hybridization. Although this may have played a role in the evolution of social hybridogenesis in the polygynous *Solenopsis* species with this reproductive system (Helms Cahan and Vinson 2003; Lacy et al. 2019), we do not expect polygyny to be critical for the evolution of worker‐loss as such loss has been described in both monogynic and polygynic species of the same genus (e.g., *Messor barbarus* and cf. *structor*; Romiguier et al. 2017). Beyond these considerations, any trait (e.g., polyandry, polygyny, or reproduction by workers) that influences kinship structure within colonies and thus modulates intracolonial conflicts has the potential to play a role in the evolution of worker‐loss. Studying the evolution of such traits and its feedback on hybridization and caste determination therefore represents an interesting avenue for future research.

More important for the evolution of worker‐loss in our model is that queens hybridize often enough. This readily happens when the propensity of queens to mate with allo‐ *versus* con‐specific males evolves (Fig. 3). In this case, sperm parasitism, worker‐loss, and social hybridogenesis emerge even in species that initially do not hybridize. Such evolution of hybridization is especially likely to occur where queens are able to recognize differences among males and choose their mates accordingly. There is, however, currently little, if any, evidence for such direct mate or sperm choice in eusocial insects (Strassmann 2001; Schwander et al. 2006; Umphrey 2006; Feldhaar et al. 2008). Alternatively, queens may be able to modulate the degree of hybridization via more indirect mechanisms, such as mating flight synchronization (Kaspari et al. 2001). Under completely random mating, hybridization can reach sufficient levels for worker‐loss to evolve in our model as long as allo‐specific males are sufficiently abundant (Fig. 2), for instance, because phenology is shared with an ecologically dominant species (Klein et al. 2017). In intermediate situations where allo‐specific males are available but scarce, the evolution of caste determination under random mating leads to a situation where queens produce both hybrid and nonhybrid workers (Fig. 2A and B). Such a scenario may be relevant to species of ants where hybrid workers has been reported but where worker‐loss has not evolved (e.g., in some North American *Solenopsis* or European *Temnothorax*; Feldhaar et al. 2008).

Whether it occurs randomly or not, hybridization requires pre‐zygotic barriers to be sufficiently low. Various mechanisms, such as secondary contacts or high dispersal ability, are known to lower these barriers (de Aguiar et al. 2009). In particular, it has been proposed that the typically low phenotypic variation among males of different ant species facilitates hybridization in this taxa (Feldhaar et al. 2008). With these considerations in mind, it is noteworthy that all known cases of social hybridogenesis have been found in ants that live in dry climates (Helms Cahan et al. 2002; Helms Cahan and Vinson 2003; Romiguier et al. 2017; Lacy et al. 2019; Kuhn et al. 2020), where the synchronicity of mating flights between species is highest due to shared dependence on punctual climatic events (Hölldobler and Wilson 1990; Feldhaar et al. 2008).

At a broader level, our results suggest that worker‐loss can readily evolve when a source of workers that is impervious to royal cheats can be exploited by queens. Besides sperm parasitism, other forms of parasitism can provide such a source of workers and have been associated with worker‐loss (Nonacs and Tobin 1992). In inquiline ants such as *Teleutomyrmex schneideri*, for instance, queens do not themselves produce workers but rather infiltrate the colony of a host and trick host workers into caring for their progeny (Hölldobler and Wilson 1990; Buschinger 2009). Like in our model, such social parasitism could be the endpoint of an arms race between queens and larvae of the same lineage, whereby increasingly caste‐biasing cheats reduce colony workforce leading queens to increasingly rely on host workers.

### CONCLUSIONS

Intracolonial conflicts are inevitably part of the social lives of nonclonal organisms. Here we have shown that such conflicts readily lead to an association between interspecific sperm parasitism and intraspecific worker‐loss via the fixation of royal cheats. This association is especially relevant to the evolution of reproductive systems that like social hybridogenesis rely on hybridization. Beyond these unorthodox systems and sperm parasitism, the fixation of royal cheats and loss of intraspecific workers may be connected to other forms of antagonistic interspecific relationships such as social parasitism. More broadly, our model illustrates how the unique conflicts that are inherent to eusocial life can lead to evolutionary arms races, with implications for elaborate reproductive systems and novel ecological interactions between species.

## AUTHOR CONTRIBUTIONS

AW, JR, and CM conceived the study. AW performed the analysis and wrote the first draft of the manuscript under the guidance of JR and CM. All authors contributed to the final version.

## DATA ARCHIVING

A Mathematica notebook that reproduces our results and a R file implementing our simulations are available here: https://zenodo.org/record/5167179.

Associate Editor: C. Moreau

## Supporting information


**Table S1**: Colonial investment in males, queens and workers.
**Figure S1**: Properties of the internal singular strategy under monoandry and low polyandry.
**Figure S2**: Polymorphism under monandry is due to positive correlational selection. A.
**Figure S3**: Non‐linear effects of investment in workers.
