# Supplementary material for: Hybridization enables the fixation of selfish queen genotypes in eusocial colonies
Source: Evol Lett. 2021 Sep 16;5(6):582–94. doi: 10.1002/evl3.253 (PMC8645202; doi:10.1002/evl3.253)
Supplement: Supplementary file 1 — Table S1: Colonial investment in males, queens and workers. Figure S1: Properties of the internal singular strategy under monoandry and low polyandry. Figure S2: Polymorphism under monandry is due to positive correlational selection. A. Figure S3: Non‐linear effects of investment in workers. [file EVL3-5-582-s001.pdf]

Appendices

A Methods

Here we describe our methods to investigate the evolutionary dynamics of: (1) the probability ω for a non-hybrid larvae to develop as a worker; and (2) the propensity η for queens to hybridize. These methods are organised as follows. First in section A.1, we present a population genetics model that describes the change in allele frequencies at a biallelic locus that determines the value of ω in larvae and of η in queens. Second (in section A.2.1), we obtain the invasion fitness of a mutant allele coding for deviant trait values in a population otherwise monomorphic for a resident allele. Then, we use this invasion fitness in section A.2 as a platform to infer the long-term adaptive dynamics of both traits (i.e. their gradual evolution under the input of rare mutations with weak phenotypic effects). Specifically, we derive the joint evolutionary equilibria of ω and η (i.e. singular values), as well as their properties (i.e. convergence stability and evolutionary stability, Dercole and Rinaldi, 2008 for textbook treatment). Finally in section A.3, we describe our individual-based simulations. A Mathematica notebook reproducing our analyses and figures is provided as a supplement here: `https://zenodo.org/record/4434257`.

A.1 Short term evolution: population genetics

A.1.1 Set-up

We consider a single locus with two alleles, a and b, that affect the expression of both ω and η in their carrier. Specifically, the probability for a larva with genotype $v \in \{aa, ab, bb\}$ to develop as a worker is ω_v, while each mate of a queen with genotype $v \in \{aa, ab, bb\}$ is allospecific with a probability η_v. To track the segregation of alleles a and b in the population, we let $p^{♀}_{aa}(t)$, $p^{♀}_{bb}(t)$, and $p^{♀}_{ab}(t)$ respectively denote the proportion of queens with genotype aa, bb and ab before mating at generation t (with $p^{♀}_{aa}(t) + p^{♀}_{bb}(t) + p^{♀}_{ab}(t) = 1$). Similarly, $p^{♂}_{a}(t)$ and $p^{♂}_{b}(t)$ respectively denote the proportion of conspecific males with haploid genotype a and b before mating at generation t (with $p^{♂}_{a}(t) + p^{♂}_{b}(t) = 1$).

A.1.2 Recurrence equations for the evolution of genotype frequencies

Our first goal is to develop recurrence equations for the frequencies of each genotype in males and females (i.e. express $p_u^{\male}(t+1)$ and $p_v^{\female}(t+1)$ in terms of $p_u^{\male}(t)$ and $p_v^{\female}(t)$ for $u \in \{a, b\}$ and $v \in \{aa, ab, bb\}$). By definition, these frequencies can be written as

$$\begin{aligned} p_u^{\male}(t+1) &= \frac{n_u^{\male}(t+1)}{n_a^{\male}(t+1) + n_b^{\male}(t+1)} \\ p_v^{\female}(t+1) &= \frac{n_v^{\female}(t+1)}{n_{aa}^{\female}(t+1) + n_{ab}^{\female}(t+1) + n_{bb}^{\female}(t+1)}, \end{aligned} \tag{A-1}$$

where $n_u^{\male}(t+1)$ is the number of males of genotype $u \in \{a, b\}$ at generation $t+1$, and $n_v^{\female}(t+1)$ the number of queens of genotype $v \in \{aa, ab, bb\}$ at generation $t+1$ in the mating pool. Under our assumption that the probability for a sexual to reach the mating pool increases with the workforce of a colony (section 2 in main text), the numbers of males and females of each genotype can be expressed as:

$$\begin{aligned} n_v^{\male}(t+1) &= x^{\male}_{aa\to v}(t) n^{\mercury}_{aa}(t) p^{\female}_{aa}(t) + x^{\male}_{ab\to v} n^{\mercury}_{ab}(t) p^{\female}_{ab}(t) + x^{\male}_{bb\to v} n^{\mercury}_{bb}(t) p^{\female}_{bb}(t) \\ n_v^{\female}(t+1) &= x^{\female}_{aa\to v}(t) n^{\mercury}_{aa}(t) p^{\female}_{aa}(t) + x^{\female}_{ab\to v}(t) n^{\mercury}_{ab}(t) p^{\female}_{ab}(t) + x^{\female}_{bb\to v}(t) n^{\mercury}_{bb}(t) p^{\female}_{bb}(t), \end{aligned} \tag{A-2a}$$

where $x^{\male}_{u\to v}(t)$ is the number of males with genotype $v \in \{a, b\}$, and $x^{\female}_{u\to v}(t)$ the number of queens with genotype $v \in \{aa, ab, bb\}$, produced by a colony founded by a queen of genotype $u \in \{aa, ab, bb\}$ at generation t. Following Reuter and Keller (2001), we assume that these numbers are proportional to the energy invested into the production of sexuals. So instead of numbers, $x^{\male}_{u\to v}(t)$ can be viewed as the investment into the production of males (of genotype $v \in \{a, b\}$) and $x^{\female}_{u\to v}(t)$ into the production of queens (of genotype $v \in \{aa, ab, bb\}$) by a colony whose queen has genotype $u \in \{aa, ab, bb\}$. Finally, $n^{\mercury}_u(t)$ is the effective workforce of a colony whose queen has genotype $u \in \{aa, ab, bb\}$ at generation t. This effective workforce is given by the sum of all types of workers present in a colony, including hybrids (with the latter weighted by their efficiency e), i.e.

$$n^{\mercury}_u(t) = \left(x^{\mercury}_{u\to aa}(t) + x^{\mercury}_{u\to ab}(t) + x^{\mercury}_{u\to bb}(t) + e x^{\mercury}_{u\to hyb}(t)\right)^{\alpha} \tag{A-2b}$$

where $x^{\mercury}_{u\to v}(t)$ is the investment into the production of workers of genotype $v \in \{aa, ab, bb, hyb\}$ (with hyb denoting hybrid genotype) made by a colony whose queen has genotype $u \in \{aa, ab, bb\}$ at generation t. The parameter $\alpha > 0$ determines the effect of the workforce on the probability

for a sexual to reach the mating pool. When $\alpha = 1$, investment in workers affects the survival of queens and males linearly (i.e. one extra unit of workforce always increases survival by the same amount). By contrast when $\alpha < 1$, investment in workers show diminishing returns. Conversely when $\alpha > 1$, investment in workers show increasing returns. For most of our analyses, we assume linear effects of the workforce ($\alpha = 1$). We relax this assumption in section B.2.3.

We specify the investments into males, $x^{♂}_{u\star v}(t)$, queens, $x^{♀}_{u\star v}(t)$, and workers, $x^{☿}_{u\star v}(t)$, in terms of model parameters in Table S1. For the sake of completeness, we do so for a model that encompasses all the effects explored sequentially in the main text, i.e. we allow for both traits ω and η to coevolve; for a finite number m of mates for each queen; and for a fraction c of a queen's brood to be produced via parthenogenesis. To read Table S1, note that the different investments made by a colony with a queen of type $u \in \{aa, ab, bb\}$ (i.e. $x^{♂}_{u\star v}(t)$, $x^{♀}_{u\star v}(t)$, and $x^{☿}_{u\star v}(t)$) depend on the types of males she has mated with. To capture this, we let $M_{u,v}$ be the random number of males of genotype $v \in \{a, b, h\}$ (where h denotes allospecific type) that a queen of genotype $u \in \{aa, ab, bb\}$ mates with. Assuming that each mate is independent from one another, these random variables follow a multinomial distribution with parameters,

$$\boldsymbol{M}_u = (M_{u,a}, M_{u,b}, M_{u,h}) \sim \text{Multinomial}\Big(m, (1-\eta_u)p^{♂}_a(t), (1-\eta_u)p^{♂}_b(t), \eta_u\Big), \qquad \text{(A-3)}$$

where m is the total number of mates; $(1-\eta_u)p^{♂}_v(t)$ is the probability that in one mating event a queen of type u mates with a conspecific male of type $v \in \{a, b\}$ (which requires that this queen does not hybridize, with probability $(1-\eta_u)$, and encounters a male of type v, with probability given by its proportion, $p^{♂}_v(t)$); and η_u is the probability that in one mating event a queen of type u mates with an allospecific male.

To get to the recurrence equations tracking the proportion of males and queens of each genotype, we first substitute the entries of Table S1 into eq. (A-2) (with $\alpha = 1$). Doing so we obtain polynomials for the numbers $n^{♂}_v(t+1)$ (for $v \in \{a, b\}$) and $n^{♀}_v(t+1)$ (for $v \in \{aa, ab, bb\}$) in terms of the random variables $M_{u,a}$, $M_{u,b}$, and $M_{u,h}$ (with $u \in \{aa, ab, bb\}$). We marginalise (i.e. take the expectation of) these polynomials over the joint probability mass function of $M_{u,a}$, $M_{u,b}$, and $M_{u,h}$ for each $u \in \{aa, ab, bb\}$, which is given by eq. (A-3). Finally, the so-obtained numbers of different types of individuals (eq. A-2) are substituted into eq. (A-1). From this operation and using the fact that $p^{♂}_a(t) = 1 - p^{♂}_b(t)$ and $p^{♀}_{aa}(t) = 1 - p^{♀}_{bb}(t) - p^{♀}_{ab}(t)$, we obtain a

Investment		Queen		
caste	type	aa	ab	bb
males	a	$x^{♂}_{aa\to a}(t) = (1-f)$	$x^{♂}_{ab\to a}(t) = \frac{1}{2}(1-f)$	$x^{♂}_{bb\to a}(t) = 0$
	b	$x^{♂}_{aa\to b}(t) = 0$	$x^{♂}_{ab\to b}(t) = \frac{1}{2}(1-f)$	$x^{♂}_{bb\to b}(t) = (1-f)$
queens	aa	$x^{♀}_{aa\to aa}(t) = f\left(c + (1-c)\frac{M_{aa,a}}{m}\right)(1-\omega_{aa})$	$x^{♀}_{ab\to aa}(t) = f(1-c)\frac{1}{2}\frac{M_{ab,a}}{m}(1-\omega_{aa})$	$x^{♀}_{bb\to aa}(t) = 0$
	ab	$x^{♀}_{aa\to ab}(t) = f(1-c)\frac{M_{aa,b}}{m}(1-\omega_{ab})$	$x^{♀}_{ab\to ab}(t) = f\left(c + (1-c)\frac{1}{2}\frac{M_{ab,a}+M_{ab,b}}{m}\right)(1-\omega_{ab})$	$x^{♀}_{bb\to ab}(t) = f(1-c)\frac{M_{bb,a}}{m}(1-\omega_{ab})$
	bb	$x^{♀}_{aa\to bb}(t) = 0$	$x^{♀}_{ab\to bb}(t) = f(1-c)\frac{1}{2}\frac{M_{ab,b}}{m}(1-\omega_{bb})$	$x^{♀}_{bb\to bb}(t) = f\left(c + (1-c)\frac{M_{bb,b}}{m}\right)(1-\omega_{bb})$
workers	aa	$x^{☿}_{aa\to aa}(t) = f\left(c + (1-c)\frac{M_{aa,a}}{m}\right)\omega_{aa}$	$x^{☿}_{ab\to aa}(t) = f(1-c)\frac{1}{2}\frac{M_{ab,a}}{m}\omega_{aa}$	$x^{☿}_{bb\to aa}(t) = 0$
	ab	$x^{☿}_{aa\to ab}(t) = f(1-c)\frac{M_{aa,b}}{m}\omega_{ab}$	$x^{☿}_{ab\to ab}(t) = f\left(c + (1-c)\frac{1}{2}\frac{M_{ab,a}+M_{ab,b}}{m}\right)\omega_{ab}$	$x^{☿}_{bb\to ab}(t) = f(1-c)\frac{M_{bb,a}}{m}\omega_{ab}$
	bb	$x^{☿}_{aa\to bb}(t) = 0$	$x^{☿}_{ab\to bb}(t) = f(1-c)\frac{1}{2}\frac{M_{ab,b}}{m}\omega_{bb}$	$x^{☿}_{bb\to bb}(t) = f\left(c + (1-c)\frac{M_{bb,b}}{m}\right)\omega_{bb}$
	hyb	$x^{☿}_{aa\to hyb}(t) = f(1-c)\frac{M_{aa,h}}{m}$	$x^{☿}_{ab\to hyb}(t) = f(1-c)\frac{M_{ab,h}}{m}$	$x^{☿}_{bb\to hyb}(t) = f(1-c)\frac{M_{bb,h}}{m}$

Table S1: Colonial investment in males, queens and workers. Each entry in the table gives the investment into one type of individuals (given caste/genotype combination; rows), in a colony led by a queen with a given genotype (columns). Each expression depends only on models parameters and genotypic values for each trait. Genotypic values for hybridization probability in queens of genotype u (η_u) do not appear explicitly but determine the distribution of the random variables $M_{u,a}$, $M_{u,b}$ and $M_{u,h}$ (eq. A-3). To see how we constructed this table, consider for e.g. the investment in queens of genotype ab in a colony led by a queen of genotype ab (fourth row, second column). First, queens can arise only from the fraction f of the brood that is diploid. Next, as the laying queen is of genotype ab, the fraction c of diploid eggs that are produced through parthenogenesis will also be of genotype ab. The fraction $(1-c)$ of diploid eggs that are fertilised through regular sex is ab with a probability that depends on the queen's mates: $(M_{ab,a} + M_{ab,b})/(2m)$ (assuming random chromosomal segregation and fertilisation, e.g. because the amount of sperm provided by each male is the same and well-mixed within a queen's spermathecae). Finally, diploid ab eggs develop into queens with probability $1 - \omega_{ab}$. The other entries of the table are derived similarly.

recurrence equation,

$$\begin{pmatrix} p_b^{\male}(t+1) \\ p_{ab}^{\female}(t+1) \\ p_{bb}^{\female}(t+1) \end{pmatrix} = \boldsymbol{F} \begin{pmatrix} p_b^{\male}(t) \\ p_{ab}^{\female}(t) \\ p_{bb}^{\female}(t) \end{pmatrix}, \tag{A-4}$$

that is characterised by a mapping $\boldsymbol{F} : [0,1]^3 \to [0,1]^3$. This recurrence is too complicated to be presented here for the general case but can straightforwardly be iterated numerically to track allelic frequency changes for given parameter values (see Mathematica notebook for e.g.).

A.2 Long-term evolution: adaptive dynamics

To gain more analytical insights, we use the recurrence eq. (A-4) to study the long term adaptive dynamics of both traits under the assumption that traits evolve via mutations that are rare and with weak additive phenotypic effects.

A.2.1 Invasion fitness of rare additive allele

An adaptive dynamics model is typically based on the invasion fitness of a mutant allele in a population that is otherwise fixed for a resident allele (i.e. the asymptotic growth rate of a mutant allele). To obtain this invasion fitness, we first introduce some notation. We denote the resident allele by a vector $\boldsymbol{z} = (\omega, \eta)$ where ω is probability that a larva homozygote for the resident allele develops into a worker, and η is the probability that a mate of queen homozygote for the resident allele is allo-specific. Similarly, the mutant allele is described by a vector $\boldsymbol{\zeta} = (\omega + \delta_\omega, \eta + \delta_\eta)$ whose first entry gives the probability that a larva homozygote for the mutant allele develops into a worker, and whose second entry is the probability that a mate of a queen homozygote for the mutant allele is allo-specific (δ_ω and δ_η thus denote the mutant effect on trait values). Assuming additive genetic effects on phenotypes, a heterozygote then expresses phenotype $(\omega + \delta_\omega/2, \eta + \delta_\eta/2)$.

To use the recurrence equations developed in the previous section, we arbitrarily set allele a as the resident and b as the mutant. The allele specific trait values (appearing in table S1 and

eq. A-3) are then replaced by:

$$\begin{aligned} \omega_{aa} &= \omega & \eta_{aa} &= \eta \\ \omega_{ab} &= \omega + \frac{1}{2}\delta_\omega & \eta_{ab} &= \eta + \frac{1}{2}\delta_\eta \\ \omega_{bb} &= \omega + \delta_\omega & \eta_{bb} &= \eta + \delta_\eta. \end{aligned} \tag{A-5}$$

Next, we use the fact that the mutant is rare so that its frequency in the population is of the order of a small parameter denoted $0 < \epsilon \ll 1$. As a rare allele can only be found in heterozygous form in a large panmictic population, the initial dynamics of a mutant allele b can be described through linear approximations of $p_b^{♂}(t+1)$ and $p_{ab}^{♀}(t+1)$ at a near-zero frequency of b (e.g. Brännström et al., 2013). In other words, eq. (A-4) can be linearised to

$$\begin{pmatrix} p_b^{♂}(t+1) \\ p_{ab}^{♀}(t+1) \end{pmatrix} = \mathbf{A}(\boldsymbol{\zeta}, \boldsymbol{z}) \begin{pmatrix} p_b^{♂}(t) \\ p_{ab}^{♀}(t) \end{pmatrix} + \mathcal{O}(\epsilon^2), \tag{A-6}$$

where $\mathbf{A}(\boldsymbol{\zeta}, \boldsymbol{z})$ is a 2×2 matrix that depends on mutant and resident phenotypes, $\boldsymbol{\zeta}$ and $\boldsymbol{z}$, and ϵ is a small parameter of the order of the frequency of the mutant b in males and queens.

The invasion fitness of the mutant phenotype, which we write as $W(\boldsymbol{\zeta}, \boldsymbol{z})$, is then given by the leading eigenvalue of $\mathbf{A}(\boldsymbol{\zeta}, \boldsymbol{z})$ (e.g. Caswell, 2000), i.e.

$$W(\boldsymbol{\zeta}, \boldsymbol{z}) = \lambda_{\max}\big(\mathbf{A}(\boldsymbol{\zeta}, \boldsymbol{z})\big), \tag{A-7}$$

where $\lambda_{\max}(\mathbf{M})$ gives the leading eigenvalue of a matrix $\mathbf{M}$. In a large population, $W(\boldsymbol{\zeta}, \boldsymbol{z})$ tells the fate of the mutant allele. If $W(\boldsymbol{\zeta}, \boldsymbol{z}) \leq 1$, then the mutant allele is purged by selection and vanishes with probability one. Otherwise if $W(\boldsymbol{\zeta}, \boldsymbol{z}) > 1$, the mutant has a non zero probability of invading the population (e.g. Brännström et al., 2013).

A.2.2 Directional selection

When mutations are rare with weak phenotypic effects, the population first evolves under directional selection whereby an advantageous mutation fixes before a new mutation arises so that the population "jumps" from one monomorphic state to another (Dercole and Rinaldi, 2008). To study these dynamics, we use the selection gradient, $\boldsymbol{s}(\boldsymbol{z})$, which is a vector pointing in the direction favoured by selection at every point $\boldsymbol{z} \in [0, 1] \times [0, 1]$ of the phenotypic space (i.e., the

space of all possible phenotypic combinations with ω and η both between 0 and 1 as they are both probabilities) . This vector is given by the marginal effect of each trait on invasion fitness, i.e.

$$\boldsymbol{s}(\boldsymbol{z}) = \begin{pmatrix} s_\omega(\boldsymbol{z}) \\ s_\eta(\boldsymbol{z}) \end{pmatrix} = \begin{pmatrix} \left.\dfrac{\partial W(\boldsymbol{\zeta}, \boldsymbol{z})}{\partial \delta_\omega}\right|_{\boldsymbol{\zeta}=\boldsymbol{z}} \\ \left.\dfrac{\partial W(\boldsymbol{\zeta}, \boldsymbol{z})}{\partial \delta_\eta}\right|_{\boldsymbol{\zeta}=\boldsymbol{z}} \end{pmatrix}, \tag{A-8}$$

where $s_\omega(\boldsymbol{z})$ and $s_\eta(\boldsymbol{z})$ give the direction of selection on ω and η respectively.

Singular strategies. A singular strategy, $\boldsymbol{z}^* = (\omega^*, \eta^*)$, is such that all selection gradients are equal to zero,

$$\boldsymbol{s}(\boldsymbol{z}^*) = \mathbf{0}. \tag{A-9}$$

A singular strategy therefore represents a potential equilibrium in the context of adaptive dynamics (Brännström et al., 2013).

Jacobian matrix and convergence stability. Whether the population evolves towards or away from a singular strategy $\boldsymbol{z}^*$ depends on the Jacobian matrix,

$$\mathbf{J}(\boldsymbol{z}^*) = \begin{pmatrix} \left.\dfrac{\partial s_\omega(\boldsymbol{z})}{\partial \omega}\right|_{\boldsymbol{z}=\boldsymbol{z}^*} & \left.\dfrac{\partial s_\omega(\boldsymbol{z})}{\partial \eta}\right|_{\boldsymbol{z}=\boldsymbol{z}^*} \\ \left.\dfrac{\partial s_\eta(\boldsymbol{z})}{\partial \omega}\right|_{\boldsymbol{z}=\boldsymbol{z}^*} & \left.\dfrac{\partial s_\eta(\boldsymbol{z})}{\partial \eta}\right|_{\boldsymbol{z}=\boldsymbol{z}^*} \end{pmatrix}. \tag{A-10}$$

Specifically, one necessary condition for a singular strategy to be an evolutionary attractor is that the greatest real part of the eigenvalues of $\mathbf{J}(\boldsymbol{z}^*)$ is negative (Leimar, 2009). Such a singular strategy $\boldsymbol{z}^*$ is said to be convergence stable. Otherwise, the population will be repelled away from $\boldsymbol{z}^*$. Even if $\boldsymbol{z}^*$ is convergence stable, it is possible for the population to evolve away from $\boldsymbol{z}^*$ when both evolving traits are genetically correlated (Leimar, 2009). A sufficient condition for a singular strategy to be an attractor is that the symmetric part of the Jacobian matrix, $(\mathbf{J}(\boldsymbol{z}^*) + \mathbf{J}(\boldsymbol{z}^*)^{\mathrm{T}})/2$, is negative-definite, in which case $\boldsymbol{z}^*$ is said to be *strongly* convergence stable (Leimar, 2009). When this is true, the population evolves towards $\boldsymbol{z}^*$, whatever the genetic correlations between both traits (i.e. independently from the statistical distribution of mutational effects on both traits).

A.2.3 Stabilising/disruptive selection.

Once the population is at an equilibrium for directional selection (i.e. a convergence stable phenotype), it either remains monomorphic under stabilising selection (when the equilibrium is evolutionary stable or uninvadable, Parker and Maynard Smith, 1990) or becomes polymorphic due to disruptive selection (when the equilibrium is not evolutionary stable or invadable, Geritz et al., 1998). When two traits are coevolving, this depends on the Hessian matrix (Phillips and Arnold, 1989, Leimar, 2009, Geritz et al., 2016),

$$\mathbf{H}(\boldsymbol{z}^*) = \begin{pmatrix} h_{\omega\omega}(\boldsymbol{z}^*) & h_{\omega\eta}(\boldsymbol{z}^*) \\ h_{\omega\eta}(\boldsymbol{z}^*) & h_{\eta\eta}(\boldsymbol{z}^*) \end{pmatrix} = \begin{pmatrix} \left.\dfrac{\partial^2 W(\boldsymbol{\zeta},\boldsymbol{z})}{\partial \delta_\omega^2}\right|_{\boldsymbol{\zeta}=\boldsymbol{z}=\boldsymbol{z}^*} & \left.\dfrac{\partial^2 W(\boldsymbol{\zeta},\boldsymbol{z})}{\partial \delta_\omega \delta_\eta}\right|_{\boldsymbol{\zeta}=\boldsymbol{z}=\boldsymbol{z}^*} \\ \left.\dfrac{\partial^2 W(\boldsymbol{\zeta},\boldsymbol{z})}{\partial \delta_\omega \delta_\eta}\right|_{\boldsymbol{\zeta}=\boldsymbol{z}=\boldsymbol{z}^*} & \left.\dfrac{\partial^2 W(\boldsymbol{\zeta},\boldsymbol{z})}{\partial \delta_\eta^2}\right|_{\boldsymbol{\zeta}=\boldsymbol{z}=\boldsymbol{z}^*} \end{pmatrix}. \tag{A-11}$$

An equilibrium $\boldsymbol{z}^*$ is uninvadable if $\mathbf{H}(\boldsymbol{z}^*)$ is negative-definite. Otherwise, selection may be disruptive and the population may experience evolutionary branching, whereby it splits among two diverging morphs (Geritz et al., 1998, Leimar, 2009, Geritz et al., 2016).

A.3 Individual-based simulations

To complement our mathematical analysis, we also performed individual based simulations (an R script implementing these is provided as a supplement here: `https://zenodo.org/record/4434257`). These simulations track a population of $N_q = 10000$ diploid queens (with $f = 0.5$, see figure legends for other parameters). Each queen is characterized by its genotype: a pair of haplotypes, each of which is given by the values of ω and η they code for (so four genotypic values in total). Simulations are initialized by setting both haplotypes of all N_q queens to the same arbitrary values (i.e. we start with a monomorphic population). Each generation of a simulation consists of the following steps:

1. **Mating**. First, queens mate. To model this process, we first compute the propensity η_i of each queen $i \in \{1, 2, \ldots, N_q\}$ to hybridize as the mean of the two relevant alleles it is carrying. Then, each queen i is mated with a number m_i of conspecific haploid males. This number m_i is drawn from a binomial distribution with m trials and success probability $(1 - \eta_i)$ (in line with eq. A-3). At the first generation, all males carry the same genetic values for ω and η as queens (i.e. the initial trait values). In subsequent generations, males

are sampled (with replacement) as single haplotypes from the $2i$ haplotypes present in the laying queens of the previous generation. Following eq. (A-2a), the probability to sample a given haplotype is weighted by the investment in workers within its colony of origin (as the investment in workers increases the probability for males to reach the mating pool).

2. **Colony development**. Each queen i settles to form a colony. We characterise each colony in two steps. First, a list is constructed that contains the $2m_i$ non-hybrid diploid female genotypes produced within each colony (i.e. the combinations of the alleles of a queen and of its conspecific mates). If thelytokous parthenogenesis is included ($c > 0$), the genotype of the queen itself is added to this list. Second, the investment in workers within each colony is calculated following equations in table S1 and eq. (A-2b). These calculations use the genetic value expressed by each of the $2m_i + 1$ non-hybrid genotype within the female progeny (characterised in the first step), as well as the proportion of the brood produced sexually and asexually (the parameter c), the proportion of conspecific and allospecific males the queen has mated with (i.e. m_i/m and $1 - m_i/m$), and the efficiency of hybrid workers (the parameter e).

3. **Next-generation queens**. To generate the next generation of queens, N_q new diploid female genotypes are sampled (with replacement) from all non-hybrid genotypes produced within each colony. Following table S1, the probability to sample a given genotype is weighted by its own genetic value of $(1 - \omega)$ and by the investment in workers within its colony of origin (as the investment in workers increases the probability for queens to reach the mating pool). Finally, each genotypic value independently mutates with probability 10^{-2}. Mutation effects are drawn independently from a normal distribution with mean 0 and standard deviation 10^{-2}. Mutated genetic values are capped between 0 and 1 to ensure that traits remain within their domain of definition.

B Analyses

Here, we present the derivations of our results summarised in the main text. These derivations are organised in the same order as they appear in the main text. As a supplement, we also provide a Mathematica (Wolfram Research, 2020) notebook that allows to follow our analyses.

B.1 Baseline model

We first explore the baseline case where females mate with a large (effectively infinite) number of mates and there is no parthenogenesis (i.e. when $m \to \infty$ and $c = 0$).

B.1.1 Independent evolution of genetic caste determination

As presented in the main text, we initially assume that hybridization η is fixed and only caste determination ω evolves. Using eq. (A-8) with $m \to \infty$ and $c = 0$, we find that the selection gradient on genetic caste determination is,

$$s_\omega(\boldsymbol{z}) = \frac{1}{6}\left(\frac{1-\eta}{\eta e + (1-\eta)\omega} - \frac{2}{1-\omega}\right). \tag{B-1}$$

Accordingly, there is a unique singular strategy ω^* for caste determination when hybridization η is fixed (i.e. ω^* such that $s_\omega((\omega^*, \eta)) = 0$),

$$\omega^* = \frac{1}{3} - e\frac{2\eta}{3(1-\eta)}, \tag{B-2}$$

which is eq. 1 of the main text.

It is straightforward to show that with hybridization fixed, the singular strategy (eq. B-2) is convergence stable (plugging eq. B-2 into the Jacobian, that is eq. A-10, for a single trait with $m \to \infty$ and $c = 0$),

$$\frac{\partial s_\omega(\boldsymbol{z})}{\partial \omega}\bigg|_{\omega=\omega^*} = -\frac{9(1-\eta)^2}{4(1+\eta(e-1))^2} < 0 \tag{B-3}$$

as well as uninvadable (plugging eq. B-2 into the Hessian, that is eq. A-11, for a single trait with $m \to \infty$ and $c = 0$),

$$\frac{\partial^2 W(\boldsymbol{\zeta}, \boldsymbol{z})}{\partial \delta_\omega^2}\bigg|_{\omega=\omega^*} = -\frac{3(1-\eta)^2}{4(1+\eta(e-1))^2} < 0. \tag{B-4}$$

Therefore, when hybridization is fixed, our analyses show that genetic caste determination will gradually evolve to the singular value eq. (B-2) and remain monomorphic for this value (which is what we observe when we simulate this scenario, fig. 2A).

B.1.2 Coevolution of genetic caste determination and hybridization

An unstable singularity. When both caste determination ω and hybridization η evolve, their trajectories under directional selection are given by the selection gradient vector,

$$\boldsymbol{s}(\boldsymbol{z}) = \begin{pmatrix} s_\omega(\boldsymbol{z}) \\ s_\eta(\boldsymbol{z}) \end{pmatrix} = \begin{pmatrix} \dfrac{1}{6}\left(\dfrac{1-\eta}{\eta e + (1-\eta)\omega} - \dfrac{2}{1-\omega}\right) \\ \dfrac{1}{1-\eta}\left(\dfrac{e}{3\left[\eta e + (1-\eta)\omega\right]} - \dfrac{1}{2}\right) \end{pmatrix} \tag{B-5}$$

(from eq. A-8 with $m \to \infty$ and $c = 0$). Solving the above for $\boldsymbol{z}^* = (\omega^*, \eta^*)$ such that $\boldsymbol{s}(\boldsymbol{z}^*) = (0, 0)$ yields a single singular strategy in two dimensional trait space,

$$\boldsymbol{z}^* = \begin{pmatrix} \omega^* \\ \eta^* \end{pmatrix} = \begin{pmatrix} e + \dfrac{e-1}{3} \\ 2 + \dfrac{1}{e-1} \end{pmatrix}, \tag{B-6}$$

which is plotted in fig. 3A against e. However, when we look at the Jacobian matrix of the system eq. (B-5) at this singular value (i.e. substitute eqs. B-5 and B-6 into eq. A-10),

$$\mathbf{J}(\boldsymbol{z}^*) = \begin{pmatrix} -\dfrac{9}{16(e-1)^2} & -\dfrac{3}{8e} \\ -\dfrac{3}{4e} & -\dfrac{(e-1)^2}{4e^2} \end{pmatrix}, \tag{B-7}$$

we see that this matrix has a negative determinant,

$$\det\left(\mathbf{J}(\boldsymbol{z}^*)\right) = -\frac{9}{64e^2} < 0 \tag{B-8}$$

so its eigenvalues cannot both be negative (since the product of the eigenvalues of a matrix is equal to its determinant). Hence the singular value $\boldsymbol{z}^*$ eq. (B-6) is not convergence stable, but rather an evolutionary repellor.

Our result that evolutionary trajectories will be repelled away from the singular value eq. (B-6)

tells us that adaptive dynamics will eventually get to the boundary of the trait space. This trait space consists of the square $[0,1] \times [0,1]$ (as both traits must be between zero and one). Two edges of this square (when $\omega = 1$ or $\eta = 1$) cannot be accessed by evolutionary dynamics as either of these trait values lead to zero fitness (as a population monomorphic for $\omega = 1$ or $\eta = 1$ produces no queen in our baseline model). We can therefore focus on dynamics along the edges $\eta = 0$ or $\omega = 0$ of the trait space, which respectively correspond to the case of hybridization avoidance and worker-loss.

Convergence to hybridization avoidance. Evolutionary dynamics will settle somewhere on the edge where hybridization is absent in the population ($\eta = 0$) only if: (1) selection on hybridization maintains it at zero (i.e. $s_\eta(\boldsymbol{z}) \leq 0$ when $\eta = 0$); and (2) selection on caste determination settles for an equilibrium ω^* (i.e. $s_\omega(\boldsymbol{z}) = 0$ for some ω^* when $\eta = 0$). From eq. (B-5), these two conditions are true when $e \leq 1/2$ and the equilibrium for caste determination is simply $\omega^* = 1/3$ (in line with eq. B-2). As established in eq. (B-3), this equilibrium is convergence stable and evolutionary stable when η is fixed.

Convergence to worker-loss. Similarly, for adaptive dynamics to converge somewhere on the edge where workers are no longer produced from regular sex ($\omega = 0$), these two conditions are necessary: (1) selection on caste determination maintains $\omega = 0$ (i.e. $s_\omega(\boldsymbol{z}) \leq 0$ when $\omega = 0$); and (2) selection on hybridization favours an equilibrium η^* (i.e. $s_\eta(\boldsymbol{z}) = 0$ for some η^* when $\omega = 0$). Substituting eq. (B-5) into these conditions, they reduce to $e \geq 1/4$ and $\eta^* = 2/3$. In addition, we see from eq. (B-5) that when $\omega = 0$,

$$\left.\frac{\partial s_\eta(\boldsymbol{z})}{\partial \eta}\right|_{\eta=2/3} = -\frac{9}{4} < 0, \tag{B-9}$$

and we further find that

$$\left.\frac{\partial^2 W(\boldsymbol{\zeta}, \boldsymbol{z})}{\partial \delta_\eta^2}\right|_{\eta=2/3} = -\frac{3}{4} < 0. \tag{B-10}$$

This tells us that the population will converge towards and remain monomorphic for $\eta^* = 2/3$ when $\omega = 0$ is fixed.

Three phase portraits. Put together, the above observations allow us to deduce that depending on the parameter e, there are three possible types of phase portraits for the adaptive

dynamics of both traits (fig. 3B-D). When $e \leq 1/4$, the singular value eq. (B-6) is outside of the trait space (or on its boundary when $e = 1/4$) and the point ($\omega = 1/3; \eta = 0$) is an evolutionary stable attractor, meaning that the population will converge towards hybridization avoidance (fig. 3B). When $e \geq 1/2$, the singular value eq. (B-6) is also outside of the trait space (or on its boundary when $e = 1/2$) and the point ($\omega = 0; \eta = 2/3$) is an evolutionary stable attractor, meaning that the population will converge towards worker-loss (fig. 3D). Finally when $1/4 < e < 1/2$, the singular value eq. (B-6) is a repellor that lies within the trait space (i.e. $0 < \omega^* < 1$ and $0 < \eta^* < 1$) and both points ($\omega = 1/3; \eta = 0$) and ($\omega = 0; \eta = 2/3$) are evolutionary stable attractors. In this case evolutionary dynamics will depend on initial values (fig. 3C).

B.1.3 Decomposition of directional selection in terms of inclusive fitness effects

The kin selection approach. In this section, we use the so-called "kin selection" or "inclusive fitness" approach to obtain the selection gradient eq. (B-5) (Taylor and Frank, 1996). This approach, which is based on invasion analyses of alleles in class-structured populations, gives the same quantitative result about directional selection than other common methods in theoretical evolutionary biology such as adaptive dynamics, population or quantitative genetics (assuming genetic variance for traits is small, e.g. Taylor and Frank, 1996, Rousset, 2004, Lehmann et al., 2016). But one particular advantage of a kin selection approach is that it immediately decomposes directional selection on mutant alleles into the sum of: (1) their direct fitness effects on the reproductive success of the individuals that express them; and (2) of their indirect fitness effects on other related individuals that can also transmit them. This decomposition allows to delineate the various forces at play in the evolution of social behaviours (Hamilton, 1964). Here, we use it to better understand the evolution towards worker-loss (and obtain fig. 3E-F).

We follow Taylor and Frank (1996)'s general method. Consider a population with mean trait values ω and η. In this population, consider a focal colony that is home to a mutant allele that codes for deviant trait values $\eta_\bullet$ in queens and $\omega_\bullet$ in larvae that carry this allele. Let ω_0 denote the mean trait value expressed by all larvae within this focal colony. Using this notation, the expected number of successful (i.e. that mate) males that are produced by the focal colony and

that carry the mutant allele is given by,

$$w_{♂} = \frac{(1-f)\big[f\left((1-\eta_{\bullet})\omega_0 + \eta_{\bullet} e\right)\big]}{(1-f)\big[f\left((1-\eta)\omega + \eta e\right)\big]}, \tag{B-11}$$

where the numerator and denominator are the total number of males produced by the focal and a random colony, respectively. For the focal colony (the numerator), $(1-f)$ is the probability that an egg is haploid (i.e. male) while the term in square brackets is the colony's investment in workers (which in our model is also the probability that a sexual reaches maturity). The denominator follows the same logic for an average colony in the population.

Similarly, the expected number of successful queens that are produced by the focal colony that carry the mutant allele is,

$$w_{♀} = \frac{f(1-\eta_{\bullet})(1-\omega_{\bullet})\big[f\left((1-\eta_{\bullet})\omega_0 + \eta_{\bullet} e\right)\big]}{f(1-\eta)(1-\omega)\big[f\left((1-\eta)\omega + \eta e\right)\big]}, \tag{B-12}$$

where $f(1-\eta_{\bullet})(1-\omega_{\bullet})$ is the number of queens produced in the focal colony and the term in square brackets is the probability that a queen survives till mating (i.e. the colony's investment in workers).

Fitness effects within a mutant colony. With the above notation, the selection gradient vector can then be computed as,

$$\boldsymbol{s}(\boldsymbol{z}) = \begin{pmatrix} s_{\omega}(\boldsymbol{z}) \\ s_{\eta}(\boldsymbol{z}) \end{pmatrix} \propto \begin{pmatrix} v_{♀}\dfrac{\partial w_{♀}}{\partial \omega_{\bullet}} + v_{♂}\dfrac{\partial w_{♂}}{\partial \omega_0} r_{\mathrm{lm}} + v_{♀}\dfrac{\partial w_{♂}}{\partial \omega_0} r_{\mathrm{lf}} \\ v_{♂}\dfrac{\partial w_{♂}}{\partial \eta_{\bullet}} r_{\mathrm{qm}} + v_{♀}\dfrac{\partial w_{♂}}{\partial \eta_{\bullet}} r_{\mathrm{qf}} \end{pmatrix}, \tag{B-13}$$

where all derivatives are evaluated at $\omega_{\bullet} = \omega_0 = \omega$ and $\eta_{\bullet} = \eta_0 = \eta$; r_{lm} is the relatedness of a female larva to a brother; r_{lf} is the relatedness of a female larva to a sister; r_{qm} is the relatedness of a queen to its sons; r_{qf} is the relatedness of a queen to its daughters; $v_{♂}$ is the reproductive value of males and $v_{♀}$ is the reproductive value of queens (all these relatedness coefficients and reproductive values are for a monomorphic population, Taylor and Frank, 1996, Rousset and Ronce, 2004, Lehmann et al., 2016). Plugging eqs. (B-11) and (B-12) into eq. (B-13) with relatedness coefficients and reproductive values corresponding to a haplodiploid system with infinite matings (i.e. $r_{\mathrm{lm}} = 1/2$, $r_{\mathrm{lf}} = 1/4$, $r_{\mathrm{qm}} = 1$, $r_{\mathrm{qf}} = 1/2$, $v_{♂} = 1/2$, $v_{♀} = 1$), we obtain expressions equivalent to eq. (B-5). But in contrast to eq. (B-5), the selection gradients in

eq. (B-13) are expressed as a sum of fitness effects of a mutant allele via a given category of individual. More specifically, the gradient $s_\omega(\boldsymbol{z})$ in eq. (B-13) is decomposed as the fitness effects of an allele coding for a mutant value of ω in larvae: on the larvae that express this allele ($v_{♀} \dfrac{\partial w_{♀}}{\partial \omega_{\bullet}}$, yellow line in fig. 3E), on their brothers ($v_{♂} \dfrac{\partial w_{♂}}{\partial \omega_0} r_{\text{lm}}$, blue line in fig. 3E), and on their sisters (i.e. queens, $v_{♀} \dfrac{\partial w_{♂}}{\partial \omega_0} r_{\text{lf}}$, red line in fig. 3E) that can also transmit the allele. Similarly, the gradient $s_\eta(\boldsymbol{z})$ in eq. (B-13) is composed of the fitness effects of an allele coding for a mutant value of η in queens: via their sons ($v_{♂} \dfrac{\partial w_{♂}}{\partial \eta_{\bullet}} r_{\text{qm}}$, blue line in fig. 3F) and daughters (i.e. queens, $v_{♀} \dfrac{\partial w_{♂}}{\partial \eta_{\bullet}} r_{\text{qf}}$, red line in fig. 3F). To construct panels E and F of fig. 3, we evaluated these five terms outlined above at every step of an evolutionary trajectory from the baseline equilibrium in absence of hybridization ($\omega = 1/3, \eta = 0$) to complete worker-loss ($\omega = 0$, $\eta = 2/3$). The evolutionary trajectory was obtained by iteration, starting from the baseline equilibrium and taking steps of size 0.001 (in units of trait space) in the direction of the selection gradient (eq. B-5).

B.1.4 Correspondence with Reuter and Keller (2001)

Here we connect our results to those of Reuter and Keller (2001), who used a kin selection approach to study the evolution of caste determination when under full queen, full larval, or mixed control (in the absence of hybridization). Our model corresponds to the case of full larval control (eq. 3 of Reuter and Keller, 2001). Our selection gradient $s_\omega(\boldsymbol{z})$, shown in eq. (B-1) with $\eta = 0$, reduces to eq. 3 of Reuter and Keller (2001) when we assume linear effects of investment in workers on colony productivity. More specifically, if we set their term $\Delta_c = \delta s/(\delta w) \times 1/f$ (their notation in their eq. 3, where Δ_c corresponds to the gain in sexual production brought by one additional worker) to

$$\Delta_c = \frac{1 - fw}{w}, \tag{B-14}$$

and assume that the population is monogynous and highly polyandrous with balanced sex-ratio (i.e. in their notation, $f = 1/2$; $g_f = 1/4$; $g_m = 1/2$; $v_f = 2$; $v_m = 1$), then we find that eq. 3 of Reuter and Keller (2001) is proportional to our selection gradient $s_\omega(\boldsymbol{z})$ (eq. B-1) with $\eta = 0$. In line with this, both yield the convergence stable equilibrium $w^* = 1/3$.

B.2 Extensions

We now consider several extensions to our baseline model.

B.2.1 Effect of finite matings

First, we relax our assumption that queens mate with an infinite number of mates (i.e. $m < \infty$).

Selection gradient. Working from eq. (A-8) with $c = 0$, we find that the selection gradient vector on caste determination ω and hybridization η under finite matings reads as,

$$\begin{aligned} \boldsymbol{s}(\boldsymbol{z}) &= \begin{pmatrix} s_\omega(\boldsymbol{z}) \\ s_\eta(\boldsymbol{z}) \end{pmatrix} \\ &= \begin{pmatrix} \frac{1}{6}\left(\frac{1-\eta}{\eta e + (1-\eta)\omega} - \frac{2}{1-\omega} + \frac{3e\eta + 2(1-\eta)\omega}{2[\eta e + (1-\eta)\omega][\eta e(m-1) + (1-\eta)\omega m + \eta\omega]}\right) \\ \frac{1}{1-\eta}\left(\frac{e}{3\left[\eta e + (1-\eta)\omega\right]} - \frac{1}{2}\right) + \frac{\omega}{6\eta}\left(\frac{1}{\eta e + (1-\eta)\omega} - \frac{m}{\eta e(m-1) + (1-\eta)\omega m + \eta\omega}\right) \end{pmatrix}. \end{aligned} \tag{B-15}$$

These gradients are complicated but we can extract relevant information by starting our analysis on the two boundaries of the trait space along which evolutionary dynamics may end up ($\omega = 0$ or $\eta = 0$). Using eq. (B-15), we ask first when is worker-loss ($\omega = 0$) stable? And second when is hybridization avoidance ($\eta = 0$) stable?

Stability of worker-loss. Worker-loss is stable only if: (1) selection maintains ω at zero (i.e. $s_\omega(\boldsymbol{z}) \leq 0$ when $\omega = 0$); and (2) selection on hybridization settles for an equilibrium η^* (i.e. $s_\eta(\boldsymbol{z}) = 0$ for some η^* when $\omega = 0$). From eq. (B-15), these two conditions reduce to

$$e \geq \frac{1}{4} + \frac{9}{8(m-1)} \tag{B-16}$$

(region above dashed line in fig. 4A) and

$$\eta^* = 2/3. \tag{B-17}$$

Note that condition (B-16) becomes impossible as $m \to 1$. This indicates that worker-loss cannot evolve under monandry in this model. For $m > 1$, it is straightforward to show that when condition (B-16) is true, the strategy $\eta = 2/3$ is both convergence stable and evolutionary stable when $\omega = 0$ (eqs. B-9 and B-10 for e.g. of the type of argument used).

Stability of hybridization avoidance. Conversely, hybridization avoidance is stable only if: (1) selection on hybridization maintains η at zero (i.e. $s_\eta(\boldsymbol{z}) \leq 0$ when $\eta = 0$); and (2) selection on caste determination in absence of hybridization settles for an equilibrium ω^* (i.e. $s_\omega(\boldsymbol{z}) = 0$ for some ω^* when $\eta = 0$). From eq. (B-15), these two conditions reduce to

$$e \leq \frac{1}{2} + \frac{1}{2}\frac{5m-1}{6m^2-m-1} \tag{B-18}$$

(region below plain line in fig. 4A) and

$$\omega^* = \frac{1}{3} + \frac{2}{3(1+3m)}. \tag{B-19}$$

Again, it is straightforward to show that when condition (B-18) holds, the strategy given by eq. (B-19) is both convergence stable and evolutionary stable when $\eta = 0$ (eqs. B-3 and B-4 for e.g. of argument).

Together, conditions (B-16) and (B-18) split the parameter space into 4 areas where both, none, or only one of the conditions are met (fig. 4A). Where condition (B-18) is met but (B-16) is not (grey region of fig. 4A), hybridization cannot evolve when rare and worker-loss cannot be maintained. We therefore focus on the three remaining cases where worker loss can emerge. Doing so, we find that there are four possible types of evolutionary dynamics.

Type 1: Evolution towards worker-loss. Where condition (B-16) is met but (B-18) is not (dark green region of fig. 4A), selection favours the emergence of hybridization and maintenance of worker-loss. In addition, it can be shown that under these conditions, there exists no singular strategy within the trait space (i.e., there exists no $\boldsymbol{z}^* = (\omega^*, \eta^*)$ such that $0 < \omega^*, \eta^* < 1$ and $\boldsymbol{s}(\boldsymbol{z}^*) = (0, 0)$, e.g. using the function Reduce[] in Mathematica, see notebook). This means that the phase portrait of evolutionary dynamics is qualitatively the same as in fig. 3D: worker-loss always evolves.

Type 2: Evolution towards worker-loss or hybridization avoidance depending on initial conditions. Where conditions (B-16) and (B-18) are met simultaneously, both worker-loss and hybridization avoidance are stable so either strategy is maintained when common (when $m \geq 5$, light green region of fig. 4A). Under these conditions, we find that there exists a singular strategy within the trait space (top row, columns $m = 5$ and $m = 6$ in fig. S1 for numerical

values, see Mathematica notebook for analytical expression). When we compute numerically the leading eigenvalue of the system's Jacobian matrix, we find that it is positive (fig. S1, second row, columns $m = 5$ and $m = 6$, dashed line), revealing that the singularity is an evolutionary repellor. Therefore the phase portrait of evolutionary dynamics is qualitatively the same as in fig. 3C: depending on initial conditions, evolutionary dynamics will lead to worker-loss or hybridization avoidance.

Type 3: Convergence stable and uninvadable intermediate strategy. Where neither condition (B-16) nor (B-18) are met, neither worker-loss nor hybridization avoidance are stable (when $m \leq 4$, blue region of fig. 4A). In this case, a singular strategy within the trait space also exists ($0 < \omega^*, \eta^* < 1$; fig. S1, top row, columns $m \in \{1, 2, 3, 4\}$ for numerical values; Mathematica notebook for analytical expression). But now, this intermediate strategy is (strongly) convergence stable as indicated by a negative leading eigenvalue of both the Jacobian matrix and its symmetric part (fig. S1, second row, columns $m \in \{1, 2, 3, 4\}$, dashed and dotted lines). When $m \in \{2, 3, 4\}$, this intermediate strategy is also uninvadable as shown by a negative leading eigenvalue of the Hessian matrix (fig. S1, second row, columns $m \in \{2, 3, 4\}$, full line). Thus, when the number of mates is between two and four ($m \in \{2, 3, 4\}$) and neither conditions (B-16) and (B-18) are met, the population converges and remains monomorphic for an intermediate strategy $0 < \omega^*, \eta^* < 1$.

Type 4: Emergence of polymorphism under monandry. When neither condition (B-16) nor (B-18) are met and $m = 1$, the convergence stable intermediate strategy is invadable (i.e., the Hessian has a positive leading eigenvalue; fig. S1, second row, column $m = 1$, dashed line). This means that once the population has converged to this intermediate strategy, it experiences frequency-dependent disruptive selection leading to polymorphism (Geritz et al., 1998, Geritz and Gyllenberg, 2005, Geritz et al., 2016). Inspection of the entries of the Hessian matrix reveals that

$$h_{\omega\eta}(\boldsymbol{z}^*)^2 - h_{\omega\omega}(\boldsymbol{z}^*)h_{\eta\eta}(\boldsymbol{z}^*) > 0 \qquad \text{(B-20)}$$

(fig. S2A, black line) and that $h_{\omega\omega}(\boldsymbol{z}^*) \leq 0$ and $h_{\eta\eta}(\boldsymbol{z}^*) \leq 0$ (fig. S2A, green and grey lines). This says that disruptive selection in our model is due to correlational selection between caste determination and hybridization (i.e. the selection that associates caste determination and hybridization, Phillips and Arnold, 1989) and only occurs because both traits are coevolving

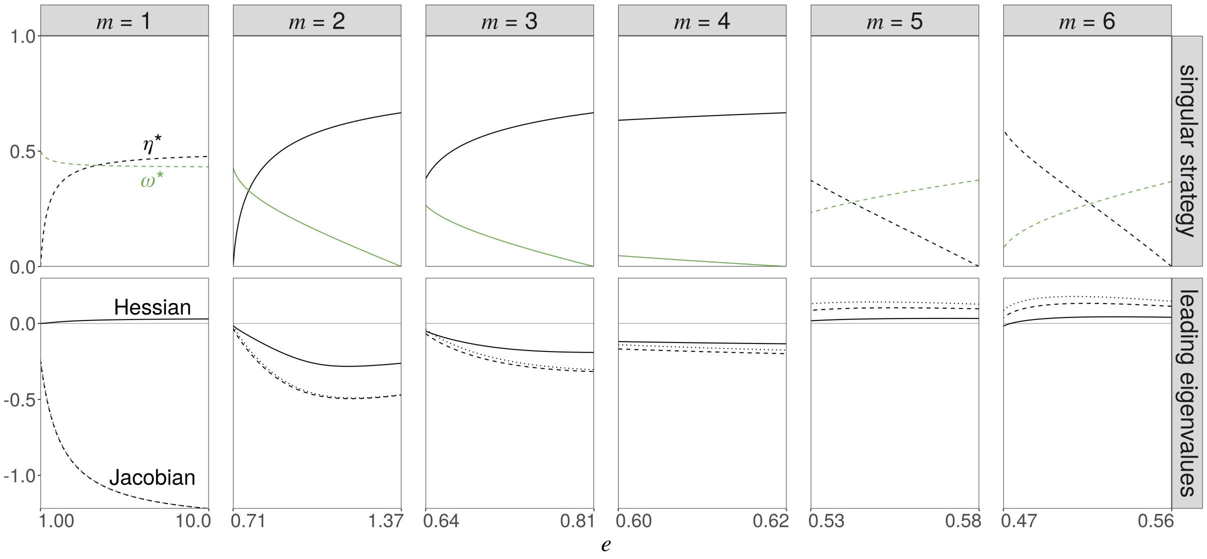

Figure S1: Properties of the internal singular strategy under monoandry and low polyandry. Each column describes the unique internal singular strategy for a specific value of m. **Top row**: value of the singular strategy (ω^* in green, η^* in black) within the range of e for which an internal strategy exists (range given by eqs. B-16 and B-18; Mathematica notebook for value of singular strategy). **Bottom row**: leading eigenvalues of the Jacobian (dashed line; for convergence stability), symmetric part of the Jacobian (dotted line; for strong convergence stability) and Hessian (full line; for evolutionary stability) matrices at the singular strategy (Mathematica notebook for calculations).

(i.e. if either trait evolves while the other is fixed, the population remains monomorphic, e.g. Mullon et al., 2018). We also find that

$$h_{\omega\eta}(\boldsymbol{z}^*) > 0 \tag{B-21}$$

(fig. S2A, blue line), which tells us that correlational selection is positive (i.e. selection favours a positive correlation between caste determination and hybridization within individuals, Phillips and Arnold, 1989). This is confirmed by individual based simulations, in which we observe the emergence of a polymorphism characterised by a positive correlation between ω and η within haplotypes (fig. 4C and fig. S2B-D).

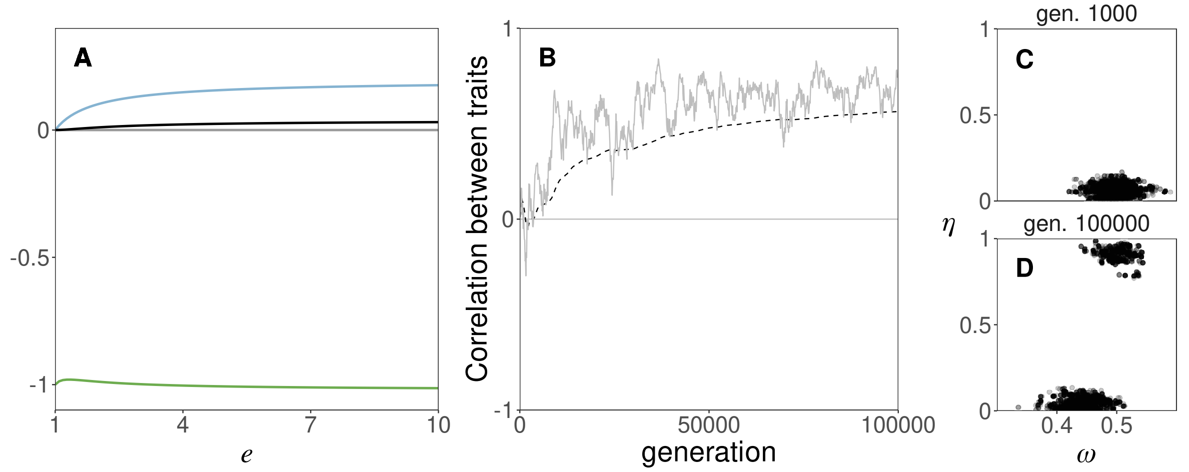

Figure S2: Polymorphism under monandry is due to positive correlational selection. **A.** Characteristics of the Hessian matrix at the internal singular strategy as a function of e for $m = 1$ (first column of fig. S1 for singular value): quadratic selection coefficient on ω ($h_{\omega\omega}(\boldsymbol{z}^*)$, in green) and on η $h_{\eta\eta}(\boldsymbol{z}^*)$, in grey); correlational selection ($h_{\omega\eta}(\boldsymbol{z}^*)$, in blue) and its relative strength ($h_{\omega\eta}(\boldsymbol{z}^*)^2 - h_{\omega\omega}(\boldsymbol{z}^*)h_{\eta\eta}(\boldsymbol{z}^*)$, in black, Mathematica notebook for calculations). **B.** Correlation between genetic values of each trait within haplotypes in a simulated population (in gray, 4000 haplotypes sampled every 100 generations to compute Pearson's correlation coefficient, same replicate as fig. 4C; cumulative mean in black dashed). **C & D** Distribution of genetic values of all haplotypes after 1000 generations (panel C) and after 100000 generations (panel D, same replicate as panel B and fig. 4C).

B.2.2 Effect of thelytokous parthenogenesis

When we allow for a fraction c of a queens brood to be produce parthenogenetically, the selection gradient (obtained from eq. A-8) is too complicated to be displayed or for singular strategies to

be found analytically. We therefore go through an invasion analysis similar to above (Appendix B.1.2 and B.2.1) and again ask: (1) under which conditions and values of ω is hybridization avoidance ($\eta = 0$) stable? and (2) under which conditions and values of η is worker-loss ($\omega = 0$) stable?

Stability of hybridization avoidance. Hybridization avoidance is stable if selection on caste determination settles for an equilibrium ω^* in the absence of hybridization (i.e. $s_\omega(\boldsymbol{z}) = 0$ for some ω^* when $\eta = 0$), and if selection on hybridization at this equilibrium maintains η at zero (i.e. $s_\eta(\boldsymbol{z}) \leq 0$ when $\eta = 0$ and $\omega = \omega^*$). These two conditions respectively reduce to,

$$\omega^* = \frac{1+c}{3+c}\left(1 + \frac{2(1-c)^2}{(c+1)\left[(1-c)^2 + (c+3)m\right]}\right), \tag{B-22}$$

and

$$e \leq \frac{3(1+c)}{2(3+c)} + \frac{(1-c)(3-c)}{2(5-c)(c+2m-1)} + \frac{4(3-c)(1-c)^2}{(5-c)(3+c)\left[(1-c)^2 + (c+3)m\right]}. \tag{B-23}$$

Condition eq. (B-23) corresponds to the area of the graph below the plain line in fig. 5A-B, where hybridization avoidance is stable. Conversely, the area above the plain line in fig. 5A-B (in blue) is where avoidance is not stable and thus where hybridization evolves.

Stability of worker-loss. Similarly, worker-loss is stable if selection on hybridization settles for an equilibrium η^* in the absence of developmental plasticity (i.e. $s_\eta(\boldsymbol{z}) = 0$ for some $0 < \eta^* < 1$ when $\omega = 0$). We find that this equilibrium reads as

$$\eta^* = \frac{2}{3}\frac{1}{1-c}\left(1 - \frac{c}{1-m}\right) \tag{B-24}$$

(fig. 5D). The equilibrium eq. (B-24) is between 0 and 1 ($0 < \eta^* < 1$) and selection at this equilibrium maintains worker-loss (i.e. $s_\omega(\boldsymbol{z}) \leq 0$ when $\omega = 0$ and $\eta = \eta^*$) when

$$e \geq \frac{1}{4} + \frac{3c}{4} + \frac{3[3 - c(12-c)]}{8(c+m-1)} - \frac{9(3-c)(1-c)c}{8(c+m-1)^2} \quad \text{and} \quad c < \frac{m-1}{3m-1}. \tag{B-25}$$

Note that condition eq. (B-25) is only possible when $m \geq 2$. It therefore does not appear in fig. 5A (which is for the case $m = 1$) but corresponds to the area above the dotted line in fig. 5B (which has $m = 2$).

Worker-loss coupled with complete hybridization. In principle, it is also possible with parthenogenesis for a population to evolve worker-loss ($\omega = 0$) with complete hybridization ($\eta = 1$) (as parthenogenesis allows the production of queens in the absence of intraspecific matings). We therefore further need to determine whether worker-loss can also be stable in the case where $\eta = 1$ (rather than for some $0 < \eta^* < 1$). We find that selection under worker-loss ($\omega = 0$) and complete hybridization ($\eta = 1$) maintains both worker-loss and complete hybridization (i.e. $s_\omega(\boldsymbol{z}) \leq 0$ and $s_\eta(\boldsymbol{z}) \geq 0$ where $\boldsymbol{z} = (\omega, \eta) = (0, 1)$) when

$$e \geq \frac{c}{1-c} \quad \text{and} \quad c \geq \frac{m-1}{3m-1}. \tag{B-26}$$

Condition eq. (B-26) corresponds to the area above the dashed line in fig. 5A-B. While condition eq. (B-25) can only be met only under polyandry ($m > 1$), condition eq. (B-26) can be met for any number of mates m. This means that the evolution of worker-loss under monandry and thelytokous parthenogenesis is always associated with complete hybridization in our model.

B.2.3 Effect of non-linear workforce productivity

Our analyses so far have assumed a linear effect of worker number on colony fitness ($\alpha = 1$ in eq. A-2b). Here we investigate how non-linear effects of the number of workers on the pre-mating survival of virgin queens and males influence our results. We restrict our exploration to the case where queens mate with an infinite number of males and do not reproduce via parthenogenesis for simplicity ($m \to \infty$ and $c = 0$). With α in eq (A-2b) as a variable, we find from eq. (A-8) that the selection gradient vector now reads as,

$$\boldsymbol{s}(\boldsymbol{z}) = \begin{pmatrix} s_\omega(\boldsymbol{z}) \\ s_\eta(\boldsymbol{z}) \end{pmatrix} = \begin{pmatrix} \dfrac{1}{6}\left(\dfrac{\alpha(1-\eta)}{\eta e + (1-\eta)\omega} - \dfrac{2}{1-\omega}\right) \\ \dfrac{1}{1-\eta}\left(\dfrac{\alpha e}{3\left[\eta e + (1-\eta)\omega\right]} - \dfrac{1+2\alpha}{6}\right) \end{pmatrix}. \tag{B-27}$$

Solving for both of these gradients to vanish simultaneously, we find that there exists a unique singular strategy,

$$\begin{cases} \omega^* = e + \dfrac{e-1}{3} \\ \eta^* = 1 + \dfrac{3e}{(e-1)(1+2\alpha)} \end{cases} \tag{B-28}$$

(fig. S3). The Jacobian matrix (eq. A-10) of the system eq. (B-27) at this singular value eq. (B-28) reads as

$$\mathbf{J}(\boldsymbol{z}^*) = \begin{pmatrix} -\dfrac{3(2+\alpha)}{16(e-1)^2\alpha} & -\dfrac{(1+2\alpha)^2}{24e\alpha} \\ -\dfrac{(1+2\alpha)^2}{12e\alpha} & -\dfrac{(e-1)^2(1+2\alpha)^3}{108e^2\alpha} \end{pmatrix}. \tag{B-29}$$

It is straightforward to show from eq. (B-29) that the singular strategy eq. (B-28) is a repellor, just as under linear effects ($\alpha = 1$, eq. B-7). This indicates that as illustrated in fig. 3, the coevolution of caste determination and hybridization under non-linear effects also lead to either hybridization avoidance or worker-loss depending on parameters and initial conditions.

We can gain further insights into the influence of non-linear effects by determining when the singular strategy eq. (B-28) is within the trait space (i.e., when $0 < \omega^*, \eta^* < 1$). We find that this is the case when

$$\frac{1}{4} < e < \frac{1+2\alpha}{4+2\alpha} \tag{B-30}$$

(light green region in fig. S3). This means that the threshold value for worker efficiency e above which worker-loss can evolve is 1/4 (as under linear effects $\alpha = 1$). Condition (B-30) further shows that the threshold for e above which worker-loss always evolves (i.e. independently from initial conditions, fig. 3D for e.g.) increases with α (dark green region in fig. S3). In other words, the evolution of worker loss is facilitated under diminishing ($\alpha < 1$, fig. S3A) and impaired under increasing returns ($\alpha > 1$, fig. S3C).

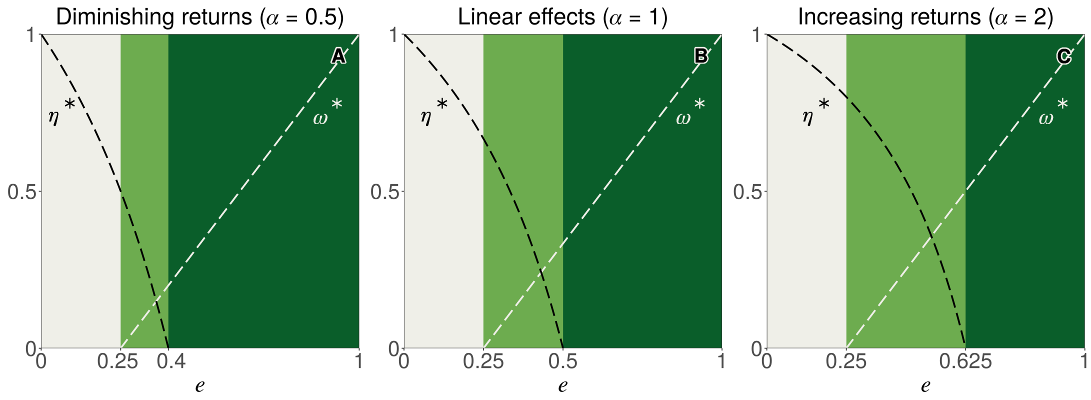

Figure S3: Non-linear effects of investment in workers. Singular values for η (in black) and ω (in white) as a function of hybrid worker efficiency e (given by eq. B-28).
